# *trans*-4,4’-Dihydroxystilbene (DHS) inhibits human neuroblastoma tumor growth and induces mitochondrial and lysosomal damages in neuroblastoma cell lines

**DOI:** 10.18632/oncotarget.17879

**Published:** 2017-05-16

**Authors:** Bhaskar Saha, Birija Sankar Patro, Mrunesh Koli, Ganesh Pai, Jharna Ray, Sandip K. Bandyopadhyay, Subrata Chattopadhyay

**Affiliations:** ^1^ Vijaygarh Jyotish Ray College, Jadavpur, Kolkata 700 032, India; ^2^ S. N. Pradhan Centre for Neuroscience, Ballygunge Science College, University of Calcutta, Kolkata 700 019, India; ^3^ Bio-Organic Division, Bhabha Atomic Research Centre, Mumbai 400085, India; ^4^ Homi Bhabha National Institute, Training School Complex, Anushakti Nagar, Mumbai 400094, India

**Keywords:** apoptosis, *trans*-4,4’-dihydroxystilbene, neuroblastoma, lysosomal membrane permeabilization, mitochondrial membrane permeabilization

## Abstract

In view of the inadequacy of neuroblastoma treatment, five hydroxystilbenes and resveratrol (Resv) were screened for their cytotoxic property against human neuroblastoma cell lines. The mechanism of cytotoxic action of the most potent compound, *trans*-4,4’-dihydroxystilbene (DHS) was investigated *in vitro* using human neuroblastoma cell lines. DHS was also tested in a mouse xenograft model of human neuroblastoma tumor. The MTT, sub-G1, annexin V and clonogenic assays as well as microscopy established higher cytotoxicity of DHS than Resv to the IMR32 cell line. DHS (20 μM) induced mitochondrial membrane permeabilization (MMP) in the cells, as revealed from JC-1 staining, cytochrome c and ApaF1 release and caspases-9/3 activation. DHS also induced lysosomal membrane permeabilization (LMP) to release cathepsins B, L and D, and the cathepsins inhibitors partially reduced MMP/caspase-3 activation. The ROS, produced by DHS activated the p38 and JNK MAPKs to augment the BAX activity and BID-cleavage, and induce LMP and MMP in the cells. DHS (100 mg/kg) also inhibited human neuroblastoma tumor growth in SCID mice by 51%. Hence, DHS may be a potential chemotherapeutic option against neuroblastoma. The involvement of an independent LMP as well as a partially LMP-dependent MMP by DHS is attractive as it provides options to target both mitochondria and lysosome.

## INTRODUCTION

Neuroblastoma is the most common solid tumor in children, and a major cause of death from childhood neoplasia. Amplification of the MYCN gene occurs in 40-50% of the high risk neuroblastoma. Patients with high risk neuroblastoma along with MYCN amplification typically show high resistance and poor therapeutic benefits [1,2]. The treatment options against neuroblastoma include chemotherapy either before surgery (neoadjuvant chemotherapy) or after surgery (adjuvant chemotherapy). In case of metastasis, chemotherapy with a combination of drugs is adopted [3]. Despite these, the prognosis of patients with advanced neuroblastoma is very poor, while majority of the chemotherapeutic agents have side effects and/ or are expensive. Hence, there is an urgent need for appropriate drug formulations for the treatment of paediatric malignancies, and the natural polyphenols may be an attractive options for this [4]. Based on epidemiological studies, consumption of vegetables and fruit-rich diets is considered to have positive impact against cancer that correlates well with their constituent polyphenolics [5,6].

The naturally occurring hydroxystilbene, resveratrol (3,4’,5-trihydroxystilbene, Resv), present in grape skins, red wines and grape juices is widely accepted as a wonder molecule because of its diverse pharmacological attributes including anticancer property [7]. Resv alone or in combination with other therapeutic drugs is reported to induce death in various human cancer cells and prevent growth factor-induced cancer progression by modulating the signalling pathways that mediate invasion, metastasis and angiogenesis [8, 9]. Regardless of these health benefits, Resv also shows different contraindicative properties [10–12]. Further, Resv can induce apoptosis in different cancer cell lines only at a significantly higher concentration [13], where its contraindicative properties are prominent. Earlier we have shown that subtle changes in the oxygenation pattern of the hydroxystilbene structure not only alleviates their toxicity but also potentiates their chemopreventive property against a host of human cancer cell lines [14, 15]. The resveratrol analog, *trans*-4,4’-dihydroxystilbene (DHS) shows remarkably higher cytotoxicity than Resv against human promyelocytic leukemia (HL-60) [16] and mouse fibroblasts, and on the proliferation and invasion of human breast cancer MCF-7 cells [17–19]. Very recently, it was found to inhibit lung cancer metastatic lesions in mice liver as well as dissemination, invasion and metastasis of lung cancer cell in a zebrafish tumor model [20]. However, nothing is known about its effect against human neuroblastoma cells.

Apoptosis induction is a preferred mode of killing the cancer cells due to less side effects and immune reactions. Many classical antitumor agents trigger caspase-mediated apoptotic cell death. Apoptosis induction typically proceeds via the intrinsic (mitochondria-caspases 9/3) and/ or the extrinsic (Fas-caspases 8/3) pathways. However, its evasion represents one of the major obstacles in anti-cancer drug development [21]. Besides mitochondria, the severely altered lysosomal volume and its trafficking as well as the levels of lysosomal hydrolases in the transformed cells can be targeted to prevent invasive growth and neoangiogenesis, and even multidrug resistance. Recently, lysosomal membrane permeabilization (LMP) of cancer cells through suitable agents has emerged as an effective strategy in cancer management [22–24].

**Figure 1:**
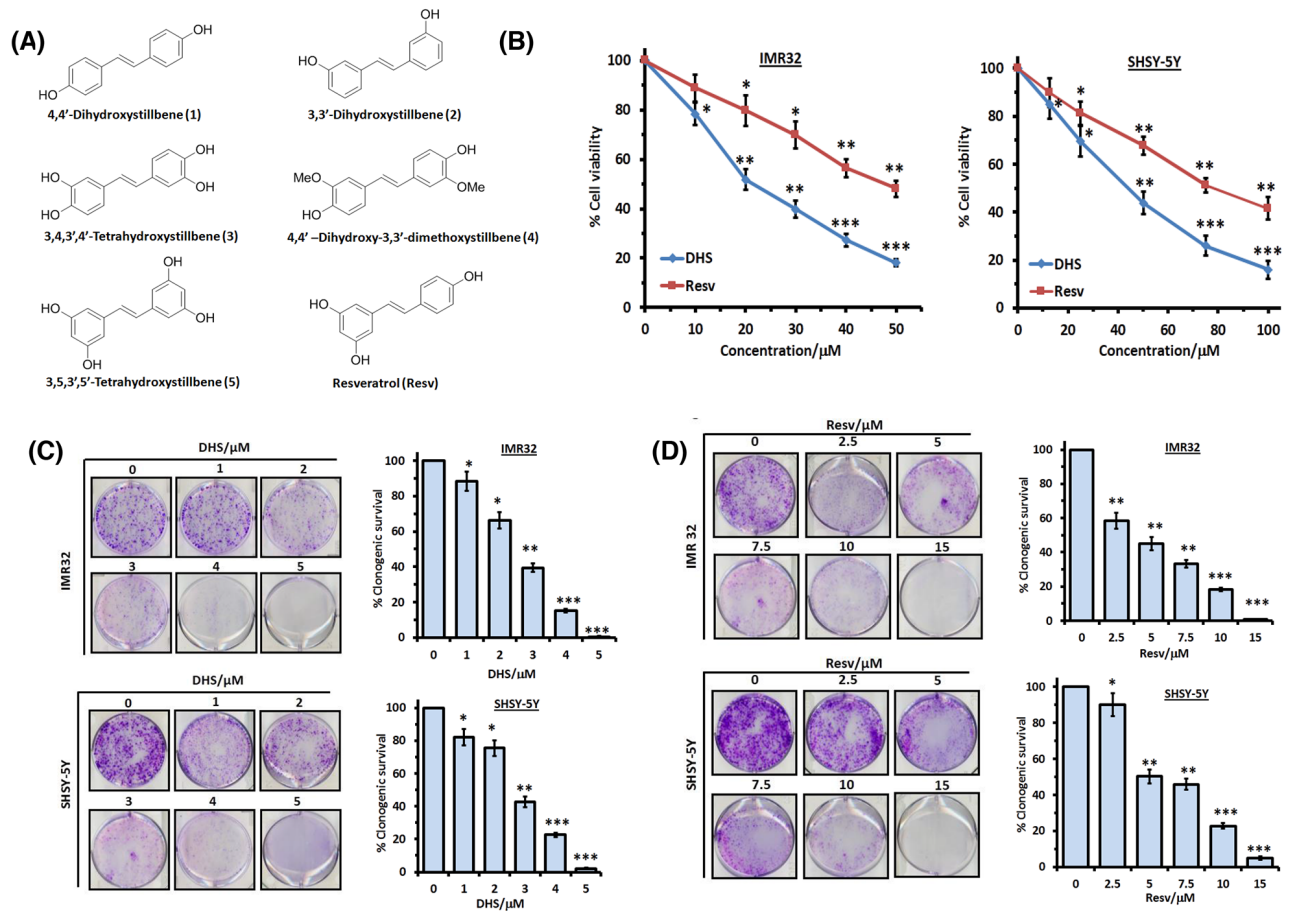
DHS is more potent than Resv in inducing death in human neuroblastoma cells **(A)** Chemical structures of the test hydroxystilbenes. **(B)** Dose-dependent cytotoxic activity of DHS and Resv against IMR32 and SHSY-5Y cell lines. The cells were incubated with vehicle (0.1% DMSO) or increasing concentrations of DHS or Resv. Cell viability at 48 h was assessed by the MTT assay. **(C)** and **(D)** Clonogenic assay. The IMR32 and SHSY-5Y cells, pre-treated with vehicle or different concentrations of DHS or Resv were grown in petri-plates for 12 days, stained with 0.5% crystal violet, and the colonies of viable cells were scanned and counted. The viable colonies were determined considering that of the untreated cells as 100 after correcting the plating efficiency. All determinations were made in five replicates in 3-4 different experiments and the values are mean ± S. E. M. ^*^p<0.05, ^**^p<0.01, ^***^p<0.001 compared to vehicle control.

Given the poor prognosis and lack of adequate treatment modality of human neuroblastoma, the present investigation was aimed to assess the potential of a series of hydroxystilbenes (compounds **1-5**) and Resv in targeting neuroblastoma, and establish the molecular mechanism of action. The chemical structures of the test compounds are shown in Figure 1A. Initially the anti-cancer property of the above hydroxystilbenes and Resv on the human neuroblastoma cell lines IMR32 and SHSY-5Y was evaluated by examining their effects on cell viability. Our results showed that DHS (compound **1**) was the most promising candidate amongst the chosen hydroxystilbenes, and the IMR32 cells were more sensitive to it. Hence, DHS and IMR32 cells were chosen for the mechanistic studies. The possible involvement of redox signalling, in particular, mitogen activated protein kinases (MAPKs) activation and mitochondrial damage in the IMR32 cells was addressed by studying the time-dependent effect of DHS in reactive oxygen species (ROS) generation, activation of the caspases, cytochrome (cyt.) c release, alteration in the BAX/BCL2 ratio, mitochondrial membrane permeabilization (MMP) and phosphorylation of specific MAPKs. To seek other alternative pathway, the ability of the DHS in inducing LMP, release of the cathepsins and BID cleavage as well as t-BID translocation was also examined. Our results clearly showed higher cytotoxicity of DHS over Resv and established that DHS induced ROS-mediated MMP as well as LMP via two parallel pathways, mediated by p38 and JNK MAPKs activation that also governed BAX and BID activation. Moreover, the DHS-induced LMP appeared to control MMP and apoptosis in the IMR32 cells, at least partially. Animal studies with the human IMR32 xenograft also showed effective inhibition of tumor growth by DHS.

## RESULTS

### DHS is more cytoxic than other hydroxystilbenes including Resv

Initially we studied the cytotoxic properties of the hydroxystilbenes **1-5** and Resv at three different concentrations (25, 50 and 75 μM) against the IMR32 (MYCN-amplified) and SHSY-5Y (non-MYCN amplified) cell lines by the MTT assay after 48 h of treatment. All the hydroxystilbenes induced significant reduction in the fractions of metabolically active cells of the tested cell lines, but were more effective against the IMR32 cell line than the SHSY-5Y cell line. In view of this, the results (Table 1) with the IMR32 cell line are shown at 25 and 50 μM of the test compounds, while that with the SHSY-5Y cell line are presented at the concentrations of 50 and 75 μM. DHS was the most active amongst the chosen compounds. The elaborate dose-dependent MTT assays, carried out with DHS revealed its IC_50_ values (at 48 h) as 21.6 ± 2.6 and 43.9 ± 3.4 μM respectively against the IMR32 and SHSY-5Y cell lines (Figure 1B). Under identical conditions, the IC_50_ values of the positive control, Resv against the above cell lines were 47.8 ± 3.3 and 78.3 ± 6.2 μM respectively (Figure 1B). A similar trend in MTT assay results was also obtained after 24 hof treatment (Supplementary Figure 1A). DHS (up to 200 μM) did not show any significant toxicity to human normal intestinal (INT-407) and kidney (HEK-293) cell lines (Supplementary Figure 1B). The vehicle (0.1% DMSO) was also nontoxic to all the cell lines. Next, we carried out the clonogenic assay to assess the effect of DHS and Resv on the above neuroblastoma cell lines. Compared to the control cells, both DHS and Resv inhibited the colony formation concentration dependently on the 12^th^ day, reflecting loss of proliferation of the cells (Figure 1C and 1D). Almost complete loss of clonogenic survival of both the cell types was observed with DHS (5 μM) and Resv (15 μM), establishing that DHS was much more cytotoxic than Resv. Besides, all the above results showed higher efficacy of DHS against MYCN amplified-IMR32 cells compared to non-MYCN amplified SHSY-5Y neuroblastoma cells. Based on these results, DHS was chosen for further studies to establish its molecular mechanism of action in MYCN-amplified IMR32 cells, a therapeutic resistant neuroblastoma cancer.

**Table 1:** Comparative cytotoxicities of the hydroxystilbenes against the human neuroblastoma cell lines^a^

Compound	% Viability loss – IMR32 cells		% Viability loss – SHSY-5Y cells	
	25 μM	50 μM	50 μM	75 μM
**1**	44.5 ± 4.2^*^	60.0 ± 4.6^**^	41.3 ± 3.4^*^	55.9 ±3.9^**^
**2**	18.2 ± 2.6	23.7 ± 2.2^*^	13.2 ± 1.6	22.8 ± 1.9^*^
**3**	35.0 ± 2.3^*^	45.4 ± 4.1^*^	38.2 ± 4.1^*^	41.2 ± 3.6^*^
**4**	37.8 ± 3.9^*^	16.2 ± 2.8	27.7 ± 1.9^*^	40.9 ± 5.1^*^
**5**	23.7 ±1.6^*^	27.2 ± 2.8^*^	17.8 ± 1.4	23.0 ± 2.2^*^
**Resv**	27.2 ± 1.8^*^	42.8 ± 4.7^*^	30.0 ± 3.6^*^	47.6 ± 5.1^**^

^a^The cells were incubated with vehicle alone or in the presence of the hydroxystilbenes under complete growth conditions. The cell viability at 48 h was assayed by the MTT method. The percentages of reduction in viable cells were calculated considering the percentage of viable cells of the vehicle-treated control as 100. All determinations were made in five replicates in 3-4 different experiments and the values are mean ± S.E.M. ^*^p<0.05, ^**^p<0.01 compared to vehicle control.

### DHS induces caspases-9- and -3-, but not caspase-8-mediated apoptosis in IMR32 cells.

**Figure 2:**
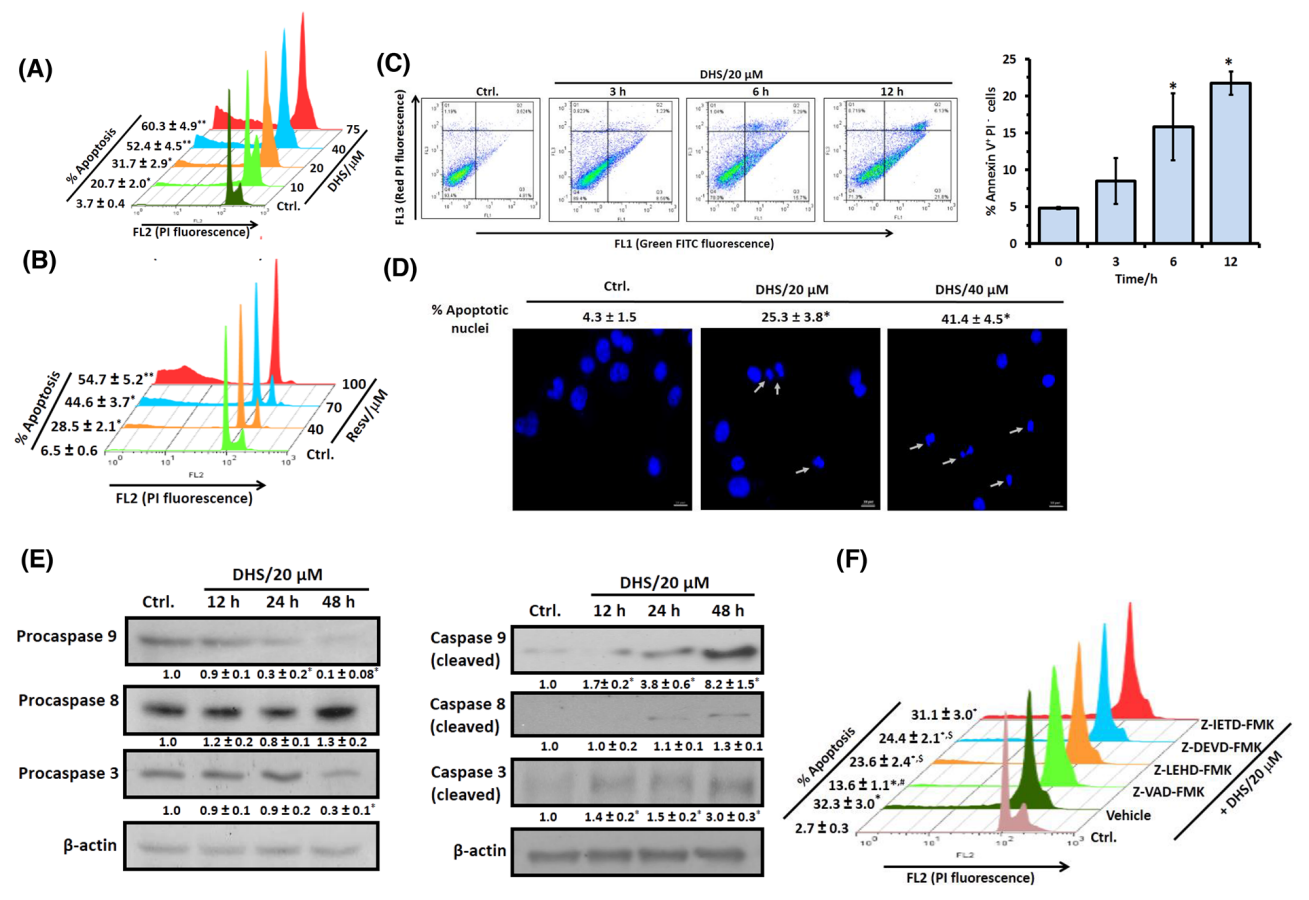
DHS induces caspase-9/caspase-3-mediated apoptosis in IMR32 cells **(A)** and **(B)** Sub-G1 population analyses. The cells were incubated with different concentrations of DHS **(A)** or Resv **(B)** for 48 h, and the sub-G1 populations analyzed by flow cytometry. **(C)** Annexin V/PI assay. The cells were incubated with DHS (20 μM) for 0-12 h, co-stained with PI and Annexin-V FITC and analyzed by flow cytometry. **(D)** Nuclear chromatin condensation. The cells were incubated with DHS (0, 20 and 40 μM) for 48 h, stained with Hoechst 33342, and observed under an Axioskop II Mot plus (Zeiss) microscope (40 × optics). The scale bar represents 10 μm, while white arrows indicate fragmented and condensed nuclei. **(E)** Expressions of the caspases. The caspases expressions in the whole cell extracts of the cells, incubated with DHS (0 and 20 μM) for different time-points were assessed by immunoblots. The protein bands were detected using a Kodak Gel-doc software and the intensity ratios of the individual bands to that of vehicle control, taken as 1 (arbitrary unit) were quantified after normalizing with respective loading controls. **(F)** Effect of the caspases inhibitors on apoptosis induction. The cells were treated with vehicle (0.1% DMSO) or pan-caspase or caspase-9/caspase-8/caspase-3-specific inhibitors (10 μM) for 1 h followed by incubation with DHS (20 μM) for 48 h, and the sub-G1 cell populations analyzed by flow cytometry. All determinations were made in duplicates for immunoblots and microscopy, and five replicates in flow cytometry analyses in 3-4 different experiments. The values are mean ± S. E. M. ^*^p<0.05, ^**^p<0.01 compared to vehicle control; ^$^*p*<0.05, ^#^*p*<0.01 compared to only DHS treatment. Representative dot plots, histograms and images are shown.

To confirm that the cell death induced by DHS was apoptotic, we looked for several apoptosis-specific parameters *viz*. (i) sub-G1 cell population, (ii) annexin V/PI staining, and (iii) nuclear DNA condensation and fragmentation in the DHS-treated cells. Treatment of the cells with different concentrations (10-75 μM) of DHSfordifferent periods (24 and 48 h) increased the sub-G1 population of the cells, both dose- and time-dependently. The increased accumulation of the DHS-treated cells in the sub-G1 phase ranged between 13.5-42.5% at 24 h (Supplementary Figure 2A) and 17.0-56.6% at 48 h (Figure 2A), compared to the respective controls. Resv treatment was less effective (Figures 2B and Supplementary Figure 2B). Under identical conditions, the positive control, cis-platin (5 μM) induced 20.5 ± 2.3% sub-G1 cell population at 48 h (data not shown).

The annexin V/PI protocol measures phosphatidylserine (PS) translocation from the inner to the outer leaflet of the plasma membrane, one of the earliest features of apoptosis. Simultaneous staining of annexin V and the non-vital dye, PI in cells allows detection and quantification of live (annexin V^-^ PI^-^), apoptotic (annexin V^+^ PI^-^) and necrotic (annexin V^+^ PI^+^) cells. Our results revealed that incubation of the IMR32 cells with DHS (20 μM) time-dependently (0-12 h) increased the annexin V^+^ PI^-^ cells. The increase was ∼11-17% at 6-12 h, compared to the vehicle control (Figure 2C). But, DHS induced only ∼0.5-5% annexin V^+^ PI^+^ cells during 3-12 h, indicating that apoptosis might be the preferred mode of cell death by DHS (Figure 2C). The chromatin condensation and fragmentation in the cells reflects the late stages of apoptosis. Presently, fluorescence microscopy of the Hoechst 33342 stained cells showed that DHS (20 and 40 μM) dose-dependently increased the number of condensed and fragmented nuclei at48 h (Figure 2D).

Next, we investigated whether caspase activation is required for the DHS-induced apoptosis. Our immunoblots showed reduced levels of pro-forms of caspase-3 and caspase-9, but almost unaltered pro-caspase-8 level after DHS (20 μM) treatment. The capase-9 pro-form was reduced by 70% and 90% at 24 h and 48 h respectively, compared to the control cells. Caspase-3 was also activated time dependently with 70% reduction of the pro-form, compared to the untreated cells at 48 h (Figure 2E, left panel). In corroboration with the above results, significantly increased levels of cleaved caspases-3 and -9, but not of caspase-8 were observed in the DHS-treated cells (Figure 2E, right panel). To further confirm, the IMR32 cells were pre-incubated with the specific caspase-9 (Z-LEHD-FMK), caspase-8 (Z-IETD-FMK) and caspase-3 (Z-DEVD-FMK) inhibitors (each 10 μM) for 1 h, followed by DHS (20 μM) treatment, andthe sub-G1 cell population analyzed at 48 h. Pre-incubation with the specific caspase-9 and caspase-3 inhibitors reduced the DHS-induced sub-G1 cell population significantly. As expected, the caspase-8 inhibitor did not show any significant effect. The pan caspase inhibitor (Z-VAD-FMK, 10 μM) also abrogated the DHS-induced apoptosis by 63.2%, but not completely (Figure 2F).

### DHS treatment elicits mitochondrial dysfunctions*.*

**Figure 3:**
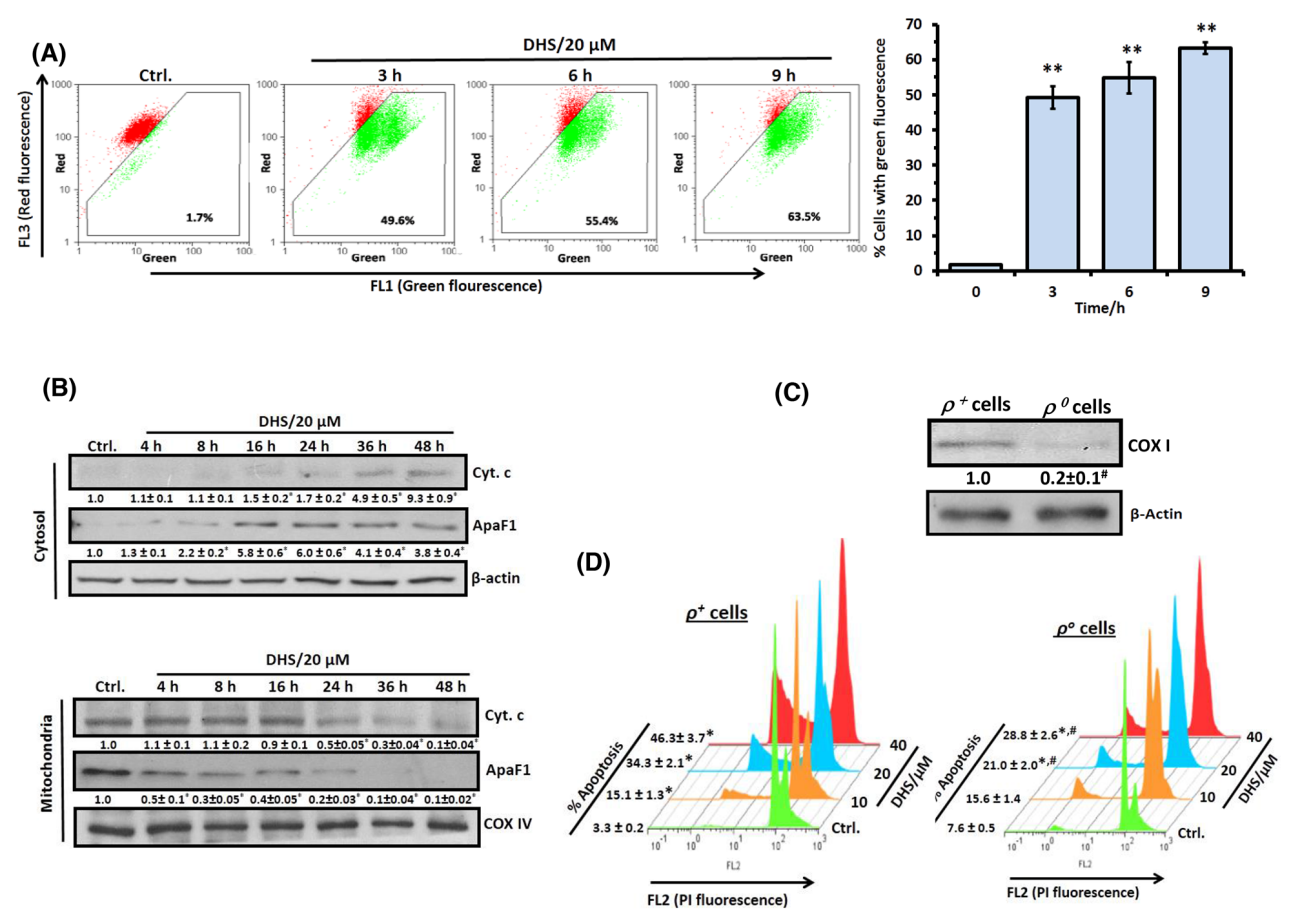
DHS permeabilizes mitochondrial membrane of IMR32 cells to induce apoptosis **(A)** Flow cytometry analyses of *ΔΨ*_m_. The cells were incubated for different periods with DHS (0 and 20 μM), stained with JC-1 for 15 min, and *ΔΨ*_m_ loss quantified by flow cytometry from the increased green fluorescense. **(B)** ApaF1and cyt. c translocation. The cytoplasmic and mitochondrial cell extracts were respectively subjected to immunoblotting, using suitable antibodies against ApaF1 and cytochrome c. **(C)** Mitochondria depletion in cells. The IMR32-*ρ*^*o*^ cells were prepared and the mitochondrial DNA deficiency was assessed from the COX I expressions in the *ρ*^*+*^ and *ρ*^*o*^ cells. The protein bands in the immunoblots were detected using a Kodak Gel-doc software and the intensity ratios of the individual bands to that of IMR32-*ρ*^*o*^ control, taken as 1 (arbitrary unit) were quantified after normalizing with respective loading controls. **(D)** Increased sensitivity of the IMR32-*ρ*^*o*^ cells to DHS treatment. The *ρ*^*+*^ and *ρ*^*o*^ cells were incubated with different concentrations of DHS (0-40 μM) for 48 h, and the sub-G1 cell populations analyzed by flow cytometry. All determinations were made in duplicates for immunoblots and five replicates for flow cytometry analyses in 3-4 different experiments. The values are mean ± S. E. M. ^*^p<0.05, ^**^p<0.01 compared to respective vehicle controls; ^#^*p*<0.01 compared to *ρ*^*+*^ cells. Representative dot plots, histograms and images are shown.

TheDHS-induced activation of caspase-9 and caspase-3 suggested the probable involvement of mitochondria-mediated intrinsic apoptotic pathway. To unravel this, we examined DHS-induced changes in mitochondria transmembrane potential (MTP; *Ψm*) and translocation of some pro-apoptotic proteins from mitochondria into cytosol. DHS treatment induced *ΔΨ*_m_ loss (∼48-62%) in the IMR32 cells during 3-9 h, as revealed by flow cytometry after JC-1 staining (Figure 3A). During 9-24 h, the *ΔΨ*_m_ loss remained unaltered (data not shown). This was associated with a time-dependent translocation of cyt. c and ApaF1 from mitochondria into the cytosol in the DHS (20 μM)-treated cells (Figure 3B). The release of cyt. c from mitochondria to cytosol started at 16 h (1.5 fold) and maximum release occurred at 48 h (9.3 fold) in response to DHS treatment. However, ApaF1 release started at 4 h (1.3 fold), reached its peak (∼6.0 fold) at 16-24 h, and thereafter reduced a little to remain steady (∼4.0 fold) at 36-48 h.

To further demonstrate the critical role of mitochondria, we analyzed the apoptosis induction in the mitochondria proficient (*ρ*^***+***^) and mitochondria depleted (*ρ*^*o*^) IMR32 cells in response to DHS treatment. The *ρ*^*o*^-IMR32 cells were characterized from the reduced expression of the mitochondria-DNA encoded protein, cytochrome oxidase I (20%, Figure 3C), an essential component of the mitochondrial respiratory chain, and reduced levels of mitochondrial DNA (30%, Supplementary Figure 3). Flow cytometry analysis revealed that the IMR32-*ρ*^*o*^ cells were more resistant to DHS at all the test concentrations, compared to the IMR32-*ρ*^***+***^ cells (Figure 3D). However, DHS was still effective against the IMR32-*ρ*^*o*^ cells, suggesting the involvement of some mitochondria-independent apoptosis.

### LMP and release of cathepsins are also involved in DHS-induced cell death

**Figure 4:**
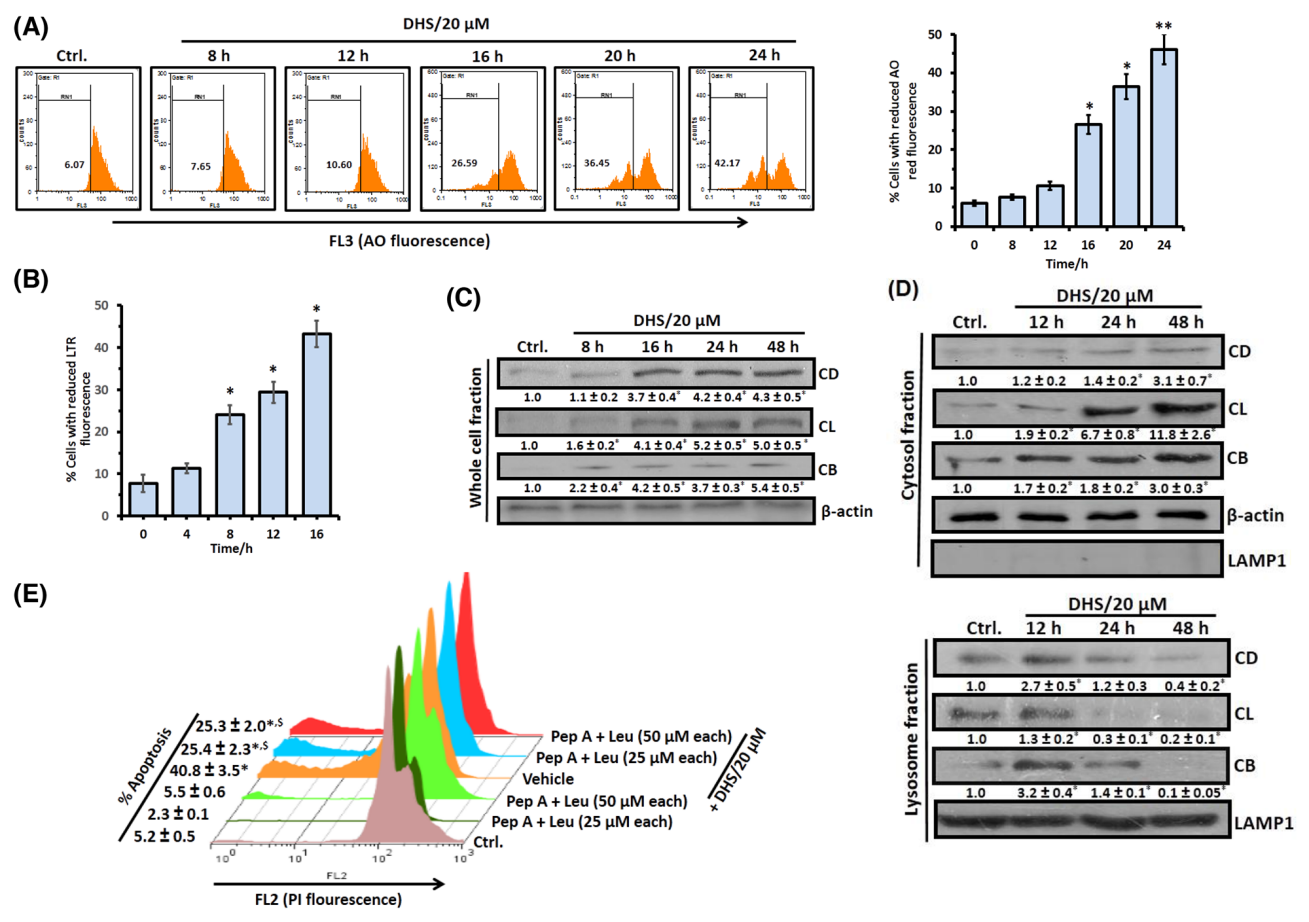
DHS induces LMP in IMR32 cells to release cathepsins that cause apoptosis **(A)** and **(B)** Flow cytometry analyses of LMP. The cells were incubated with DHS (20 μM) for 0-24 h, stained with AO or LTR and analyzed by flow cytometry. The % of cells showing reduced red fluorescence (FL3 channel) was used to quantify LMP. **(C)** and **(D)** Expressionsof cathepsins B, L and D and their translocations into cytosol. The cells were incubated for 0-48 h with DHS (0 and 20 μM) and the whole cell, cytoplasmic and lysosomal extracts were subjected to immunoblotting, using suitable antibodies against the mature forms of CB, CL and CD. The protein bands were detected using a Kodak Gel-doc software and the intensity ratios of the individual bands to that of vehicle control, taken as 1 (arbitrary unit) were quantified after normalizing with respective loading controls. **(E)** Effect of cathepsins inhibitors on apoptosis. The cells were treated with vehicle (0.1% DMSO) or Pep A-Leu mixture for 1 h followed by incubation with DHS (0 and 20 μM) for 48 h, and the sub-G1 cell populations analyzed. All determinations were made in duplicates for immunoblots and five replicates for flow cytometry analyses in 3-4 different experiments. The values are mean ± S. E. M. ^*^p<0.05, ^**^p<0.01 compared to vehicle control; ^$^*p*<0.01 compared to only DHS treatment. Representative histograms and images are shown.

Since controlled LMP has emerged as a significant inducer of MMP and apoptosis [22, 25], we also examined if DHS treatment affects lysososmal function/integrity and induces LMP in the IMR32 cells. For this, we investigated its effect on lysosomes using the lysosomotropic fluorochrome, acridine orange (AO) and the acidophilic dye, LysoTracker Red (LTR). Treatment of the cells with DHS resulted in a substantial time-dependent decrease (Figure 4A and 4B) in acidic vesicular organelles as determined from the percentage of cells with reduced red fluorescence of AO (12-24 h) and LTR (4-16 h). Subsequently, LMP induction by DHS was confirmed from release of the lysosomal cathepsins, by immunoblots. Translocation of cathepsin proteases and other hydrolytic enzymes to the cytosol is a direct consequence of LMP. The whole cell extract of the untreated cells exhibited very low levels of the mature forms of cathepsin B (CB), cathepsin L (CL) and cathepsin D (CD). But DHS (20 μM) treatment led to a time-dependent increase in the CB and CL levels (8-48 h) and CD level (16-48 h) (Figure 4C). We observed insignificant changes in the levels of active cathepsins in the whole cell extracts (WCEs) of the DHS-treated *vis-à-vis* control cells at the initial time points (0-6 h, data not shown).

Next, we examined the expressions of CB, CL and CD in the cytosolic and lysosomal fractions of the cells, treated with DHS for 12-48 h. There was a progressive increase of CB, CL and CD levels in the cytosol along with decrease in their lysosomal levels at 12-48 h after DHS treatment, confirming their translocation from the lysosome to the cytosol (Figure 4D). The involvement of these three cathepsins in the DHS-induced apoptosis in the IMR32 cells was confirmed using suitable inhibitors. Pre-treatment of cells with a mixture of inhibitors *viz*., pepstatin A (Pep A, specific for the aspartic type proteases like CD) and leupeptin (Leu, specific for cysteine type proteases such as CB and CL) significantly reduced the DHS-induced sub-G1 population (Figure 4E). However, none of these inhibitors individually showed much effect (Supplementary Figure 4).

### DHS modulates BCL-2 family of proteins to induce apoptosis in IMR32 cells

**Figure 5:**
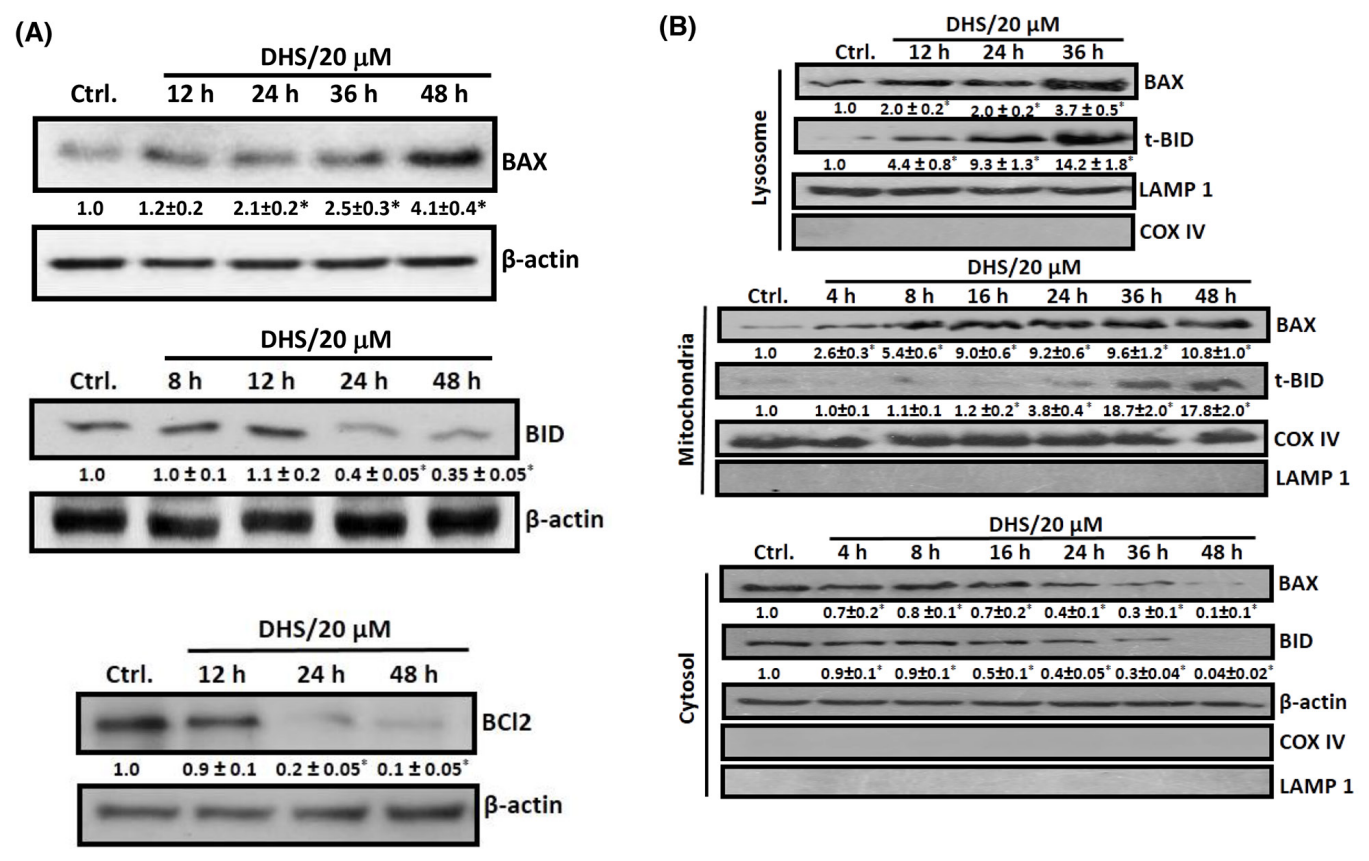
DHS increases BAX/BCL2 ratio and induces BID cleavage in IMR32 cells **(A)** Upregulation of BAX expression and BID cleavage and downregulation of BCL2 in the whole cell extract. **(B)** Translocation of BAX and t-BID into lysosome and mitochondria. The cells were incubated for different periods with DHS (0 and 20 μM) and the whole cell, cytoplasmic, lysosomal and mitochondrial extracts were subjected to immunobloting, using suitable antibodies against the above proteins. The protein bands were detected using a Kodak Gel-doc software and the intensity ratios of the individual bands to that of vehicle control, taken as 1 (arbitrary unit) were quantified after normalizing with respective loading controls. All determinations were made in duplicates in 3-4 different experiments, and the values are mean ± S. E. M. ^*^p<0.05 compared to the corresponding vehicle controls. Representative images are shown.

The BCL-2 family members, especially BAX, BCL2, BAD and BID are crucial for the MMP- and LMP-induced apoptosis [26–27]. Hence, we checked the cellular and sub-cellular (cytosolic, mitochondrial and lysosomal) expressions and translocation of some of these proteins in the DHS-treated cells. DHS treatment drastically increased the BAX expression by 1.1 to 3.1 fold, but reduced that of BCL2 (80-90%) and BID (∼60%) in the WCE, compared to the control during 24-48 h (Figure 5A). The effect on BAX, BCL2 and BID was initiated during 12-24 h. This was associated with a significant time-dependent lysosomal accumulation of BAX *viz*. 2.0-fold, 2.0-fold and 3.7-fold, and t-BID *viz*. 4.4-fold, 9.3-fold and 14.2-fold at 12, 24 and 36 h, following DHS treatment (Figure 5B), but not at early time points (data not shown). More importantly, DHS treatment induced an early (4 h) translocation of BAX, but delayed (24 h) t-BID translocation to the mitochondria (Figure 5B). Thereafter, the levels of both the proteins in mitochondria increased time-dependently. In corroboration with the lysosomal and mitochondrial translocation results, corresponding depletions of the cytosolic BAX (4 h onwards) and BID expressions (8 h onwards) were observed in the DHS-treated cells (Figure 5B).

**Figure 6:**
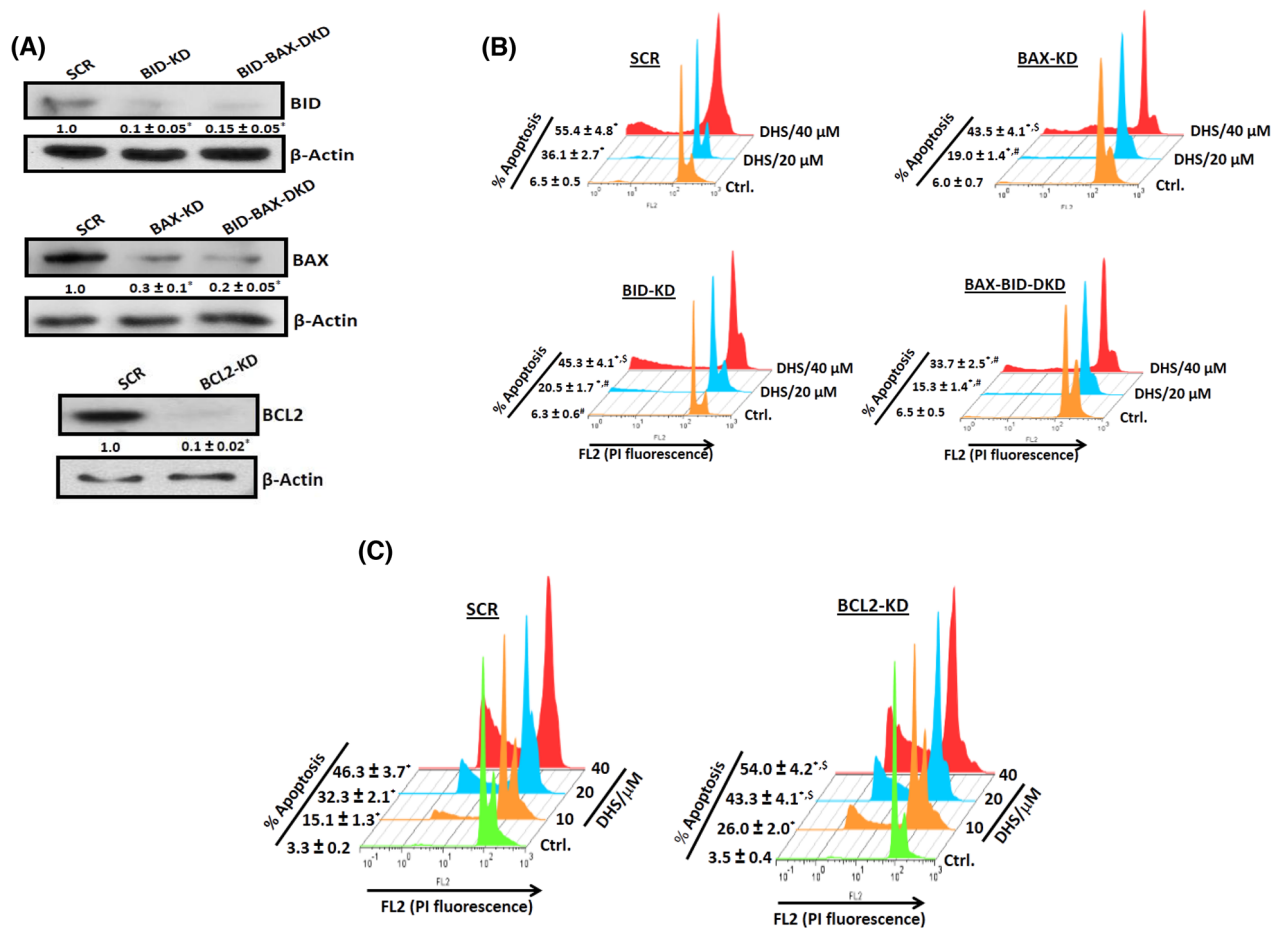
The BCL-2 family proteins are crucial for the DHS-induced apoptosis in IMR32 cells **(A)** Silencing the BCL-2 family proteins. **(B)** and **(C)** Effect of BCL-2 family proteins on the DHS-induced apoptosis. apoptosis. The SCR and KD cells were generated by siRNA/shRNA techniques and the levels of the target proteins assessed by immunoblotting the respective whole cell extracts, using suitable antibodies against the above proteins. The protein bands were detected using a Kodak Gel-doc software and the intensity ratios of the individual bands to that of respective vehicle control, taken as 1 (arbitrary unit) were quantified after normalizing with respective loading controls. The respective SCR and KD cells were incubated with different concentrations (0-40 μM)of DHS for 48 h, and the sub-G1 populations analyzed by flow cytometry. The protein bands were detected using a Kodak Gel-doc software and the intensity ratios of the individual bands to that of respective vehicle control, taken as 1 (arbitrary unit) were quantified after normalizing with respective loading controls. All determinations were made in duplicates for immunoblots and five replicates for flow cytometry analyses in 3-4 different experiments. The values are mean ± S. E. M. ^*^p<0.05 compared to vehicle control; ^$^*p*<0.05, ^#^*p*<0.01 compared to respective SCR cells. Representative images and histograms are shown.

To probe the roles of BAX, BCL2 and BID in the DHS-mediated apoptosis, we generated IMR32 cells, depleted with BAX (70% depletion), BID (90% depletion), BAX-BID together (∼85% depletion) and BCL2 (90% depletion) (Figure 6A). The cells transfected with siRNA/shRNA, possessing scrambled sequence served as the respective SCR controls. The gene-specific siRNA/shRNA transfected cells were named as BAX-KD (BAX-knock down), BID-KD (BID-knock down), BAX-BID-DKD (BAX-BID-double knock down) respectively. Next, we assessed the response of all these cells to DHS treatment. Our results showed that DHS treatment (20 μM, 48 h) induced ∼30, 13, 14 and 9% apoptotic sub-G1 population in SCR, BAX-KD, BID-KD and BAX-BID-DKD cells respectively (Figure 6B). A similar effect was also observed with DHS (40 μM). On the other hand, the BCL2-KD cells were more sensitive to DHS (20 μM) than the SCR cells, showing increased sub-G1 population at 48 h (∼29% in SCR vs 40% in BCL2-KD, Figure 6C). Similar effects were also observed with DHS (10 and 40 μM).

### DHS triggers ROS generation to induce apoptosis via MAPKs activation

**Figure 7:**
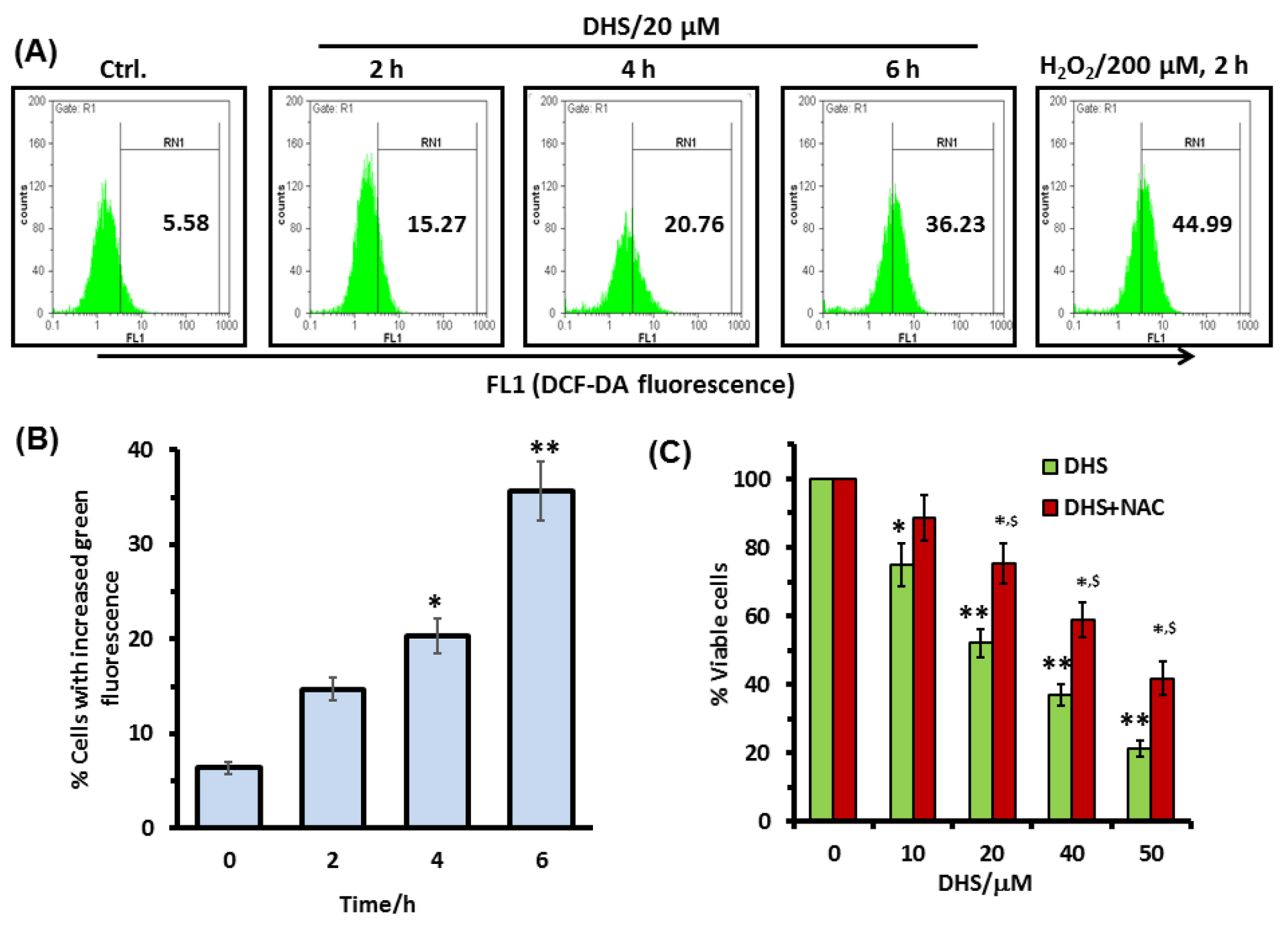
Intracellular ROS is involved in the DHS-induced cytotoxicity in IMR32 cells **(A)** and **(B)** ROS generation by DHS as revealed by flow cytometry. Cells were treated with DHS (0 and 20 μM) for different time periods and the ROS levels measured by the H2DCF-DA assay. **(C)** Effect of NAC on cell viability after DHS treatment. The cells were pretreated with NAC (5 mM) or sham medium for 1 h, further incubated for 48 h with DHS and the cell viability assayed by MTT reduction method. All determinations were made in five replicates in 3-4 different experiments. The values are mean ± S. E. M. ^*^p<0.05, ^**^p<0.01 compared to vehicle control; ^$^*p*<0.05 compared to only DHS treatment. Representative histograms are shown.

Many chemotherapeutic agents have profound effects on the cellular redox status, and generation of excess ROS acts as a signal for apoptosis induction through the activation of the MAPK signaling pathways [28, 29]. Hence, we used the H2DCF-DA assay to see if ROS is a potential factor in the DHS-induced toxicity to the IMR32 cells. Treatment of the cells with DHS (20 μM) time-dependently increased the ROS generation (Figure 7A and 7B). DHS treatment increased the ROS levels by 9.7-30.6% at 2-6 h, compared to the control cells. Under similar conditions, the positive control, H_2_O_2_ (200 μM) increased the ROS level by 39.4% at 2 h. Subsequently, the role of ROS in the DHS-induced cytotoxicity was probed by carrying out the MTT assay of the cells, pre-incubated with the cell permeable antioxidant, N-acetyl cystine (NAC) (5 mM) for 1 h, followed by DHS treatment. Compared to the only DHS-treated cells, those pre-incubated with NAC showed increased cell viability, at all the test concentrations of DHS. The results were similar when the MTT assays were carried out at 24 h and 48 h after the DHS treatment (Supplementary Figures 5 and 7C respectively).

**Figure 8:**
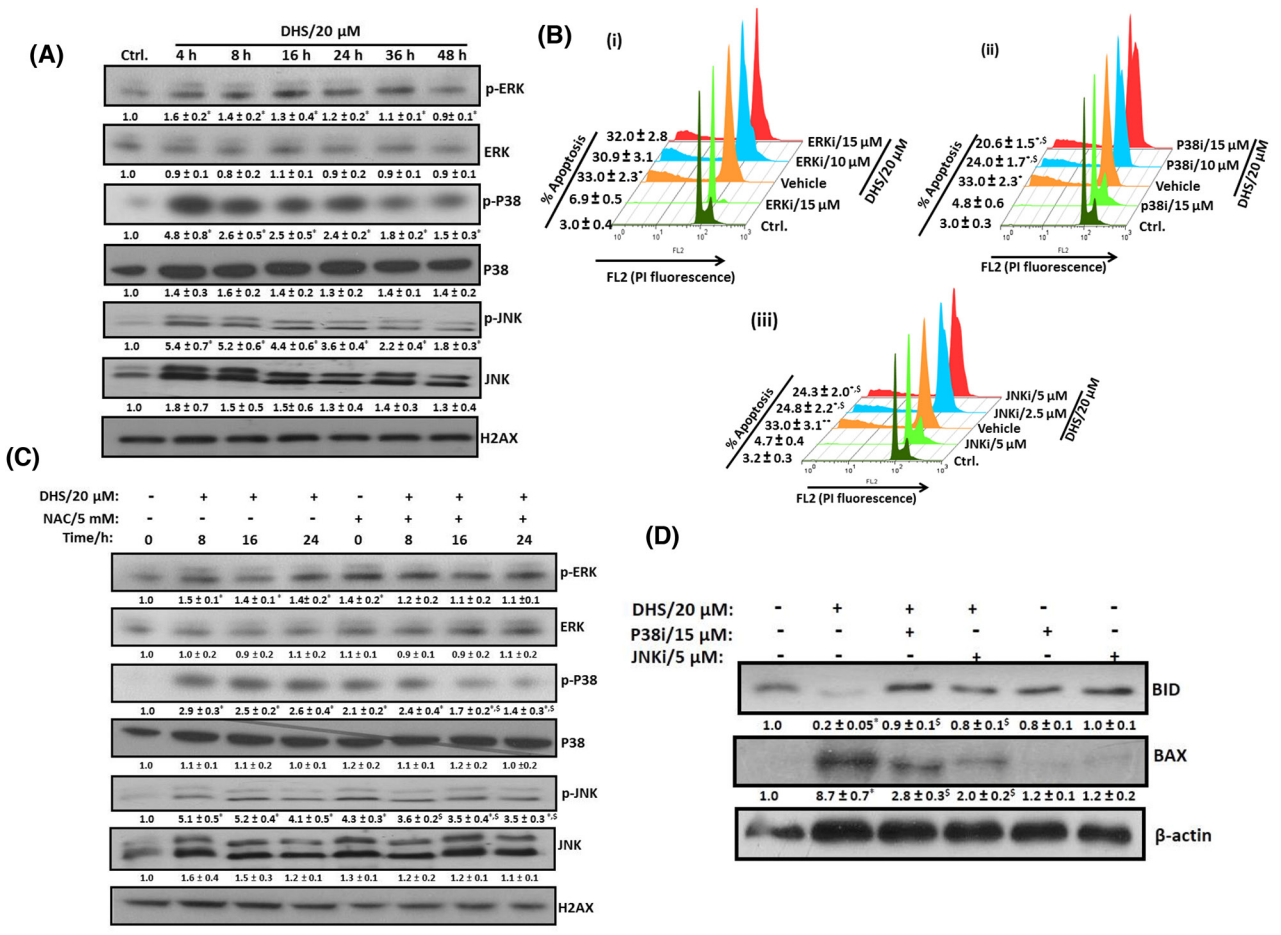
DHS-induced ROS activates p38 and JNK to modulate the BAX and BID levels and cause apoptosis in IMR32 cells **(A)** Activation of p38 and JNK by DHS. **(B)** Effects of specific MAPK inhibitors on apoptosis induction by DHS. **(C)** Effect of NAC on p38 and JNK activation by DHS. **(D)** Effect of p38 and JNK inhibitors on BAX expression and BIDcleavageby DHS. The cells were treated with DHS (0 and 20 μM) for different time periods and the MAPKs expressions analyzed by immunoblots. Similar experiments were also carried out by incubation with NAC (5 mM) for 1 h followed by treatment with DHS (0 and 20 μM) for different periods. The MAPKs phosphorylations and expressions in the cells treated with DHS (0 and 20 μM), NAC alone or in combination were analyzed by immunoblots. The sub-G1 populations of the cells treated with DHS (0 and 20 μM), the MAPKs inhibitors alone or in combination were analyzed by flow cytometry at 48 h, and the BAX and BID expressions analyzed by immunoblots. The whole cell extracts were used for all the immunoblots. The protein bands were detected using a Kodak Gel-doc software and the intensity ratios of the individual bands to that of vehicle control, taken as 1 (arbitrary unit) were quantified after normalizing with respective loading controls. All the determinations were made in duplicates for immunoblots and five replicates for flow cytometry analyses in 3-4 different experiments. The values are mean ± S. E. M. ^*^p<0.05 compared to vehicle control; ^$^*p*<0.05 compared to respective DHS treatments. Representative images and histograms are shown.

To probe the role of MAP kinase pathway in the DHS-mediated cytotoxicity to the IMR32 cells, we examined the effect of DHS on the phosphorylation of p38, ERK 1/2, and JNK MAPKs in the cells. As shown in Figure 8A, DHS (20 μM) treatment significantly increased the phosphorylation of p38 and JNK MAPKs within 4 h. The activation of p38 and JNK MAPKs were sustained up to 36 h and 48 h respectively. The p-ERK 1/2 level was, however, reduced at 16 h, after a small rise at 4 h. DHS did not alter the expression levels of non-phosphorylated forms of the MAPKs. To further confirm the signaling pathway, specific inhibitors of MAPKs such as SB203580 (p38 inhibitor; p38i), U0126 (ERK 1/2 inhibitor; ERKi), and SP600125 (JNK inhibitor; JNKi) were also employed. As expected, amongst the inhibitors, p38i (10 and 15 μM) and JNKi (2.5 and 5 μM), but not ERKi (10 and 15 μM) reduced the sub-G1 population of the DHS (20 μM)-treated cells (Figures 8B, i-iii). On its own, NAC (5 mM) increased the basal levels of phosphorylation in the tested MAPKs. However, it significantly reduced the p-p38 and p-JNK levels of the DHS-treated cells at all the chosen time points (Figure 8C). Next, we examined the up-stream role of the activated p38 and JNK MAPKs in BAX and BID activation by DHS. Pretreatment with p38i (15 μM) and JNKi (5 μM) individually, prevented the DHS-induced BID cleavage and BAX expression (Figure 8D).

### BAX and BID activation is important for DHS-mediated LMP that partly regulates MMP induction

**Figure 9:**
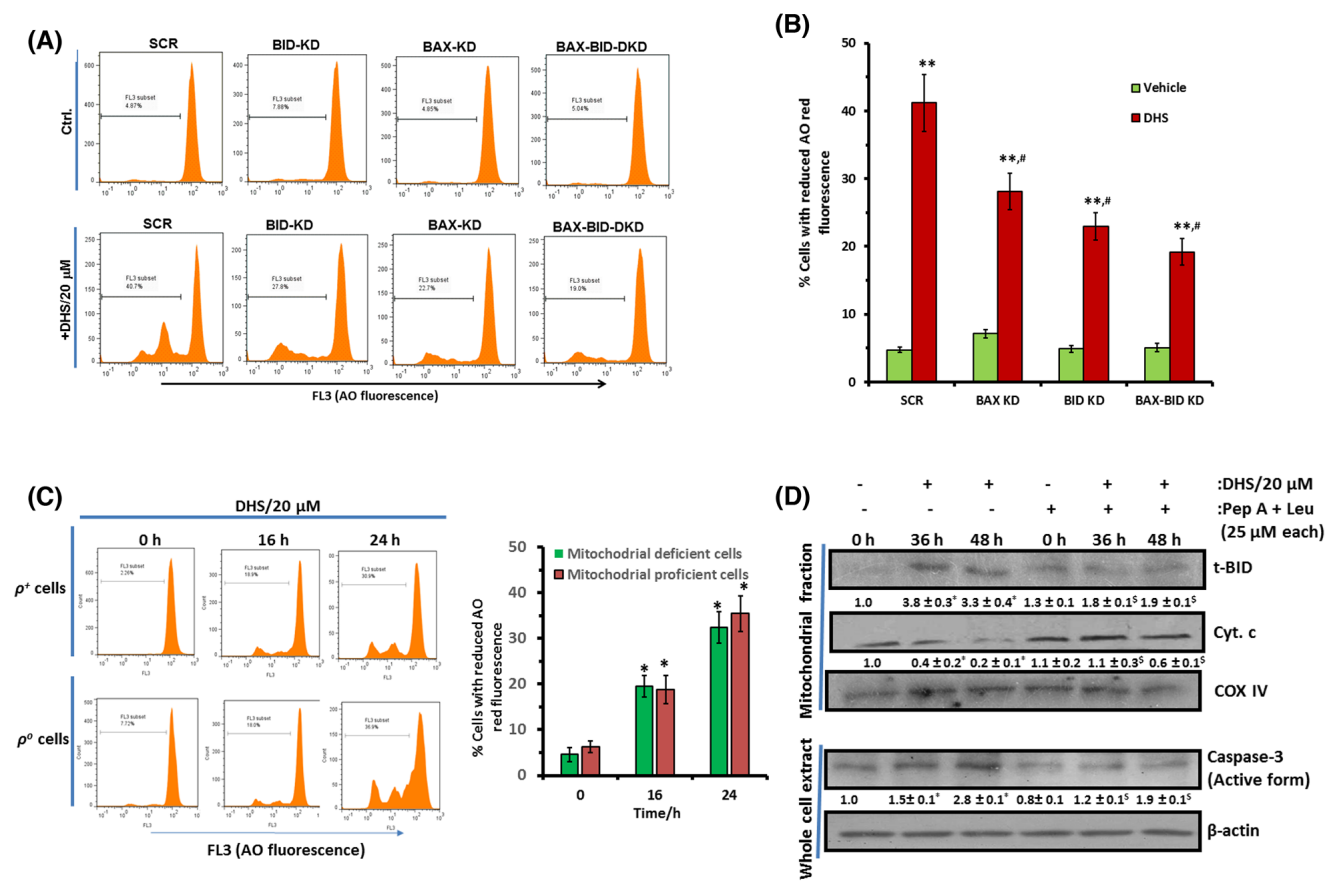
DHS-induced BAX and BID activation leads to LMP that partially controls MMP in IMR32 cells **(A)** and **(B)** LMP induction by DHS in the SCR, and BAX/BID/ BAX-BID-silenced cells and its quantification. **(C)** Effect of mitochondrial deficiency on DHS-induced LMP. **(D)** Effect of cathepsins inhibitors on mitochondrial accumulation of t-BID and cyt. c release and caspase-3 activation by DHS. The SCR controls and KD cells as well as the *ρ*^*+*^ and *ρ*^*o*^ cells were incubated with DHS (20 μM), and the induced LMP was quantified by flow cytometry after staining with AO. The t-BID and cyt. c levels in the mitochondrial extracts and active caspase-3 expressions in the whole cell extracts of the untreated, DHS-, cathepsins inhibitors- and DHS + cathepsins inhibitors-treated cells were analysed by western blotting. Cells were preincubated with cathepsin inhbitors for 1 h, prior to DHs treatment for these experiments. The protein bands were detected using a Kodak Gel-doc software and the intensity ratios of the individual bands to that of vehicle control, taken as 1 (arbitrary unit) were quantified after normalizing with respective loading controls. All determinations were made in duplicates for immunoblots and five replicates for flow cytometry analyses in 3-4 different experiments. The values are mean ± S. E. M. ^*^p<0.05, ^**^p<0.01 compared to respective vehicle controls; ^$^*p*<0.05 compared to DHS treatment; ^#^*p*<0.05 compared to DHS-treated SCR-cells. Representative images and histograms are shown.

To establish a direct correlation between BAX and BID activation and LMP by DHS, we measured the LMP (by AO assay) in the untreated and DHS (20 μM)-treated SCR, BAX-KD, BID-KD and BAX-BID-DKD cells, by flow cytometry. The untreated KD cells did not show any change in LMP compared to the control SCR cells. However, DHS induced significantly high level of LMP in the SCR cells at 24 h. This was significantly lesser in the BAX-KD, BID-KD and BAX-BID-DKD cells (Figure 9A and 9B). To ascertain any inter-dependence between MMP and LMP, we examined the effects of DHS (20 μM) in inducing (1) LMP in the mitochondria deficient (*ρ*^*o*^-IMR32) and mitochondria proficient (*ρ*^*+*^-IMR32) cells, and (2) MMP in IMR32 cells in the presence of cathepsin inhibitors. As shown in Figure 9C, mitochondrial deficiency did not significantly alter the DHS-mediated LMP at 16 h and 24 h. However, the cathepsin inhibitors (Pep A + Leu combination) significantly abrogated the DHS-induced mitochondrial accumulation of t-BID at 36 h and 48 h in the IMR32 cells (Figure 9D). Expectedly, presence of Pep A + Leu combination also reduced the DHS-induced mitochondrial release of cyt. c and caspase-3 activation significantly at the same time points (Figure 9D). We chose the time points based on appreciable LMP induction (16-24 h) (Figure 4A) as well as t-BID accumulation and cyt. c release (36-48 h) from mitochondria in response to DHS treatment (Figure 3B and 5B).

### DHS reduces neuroblastoma tumor burden in SCID mice

**Figure 10:**
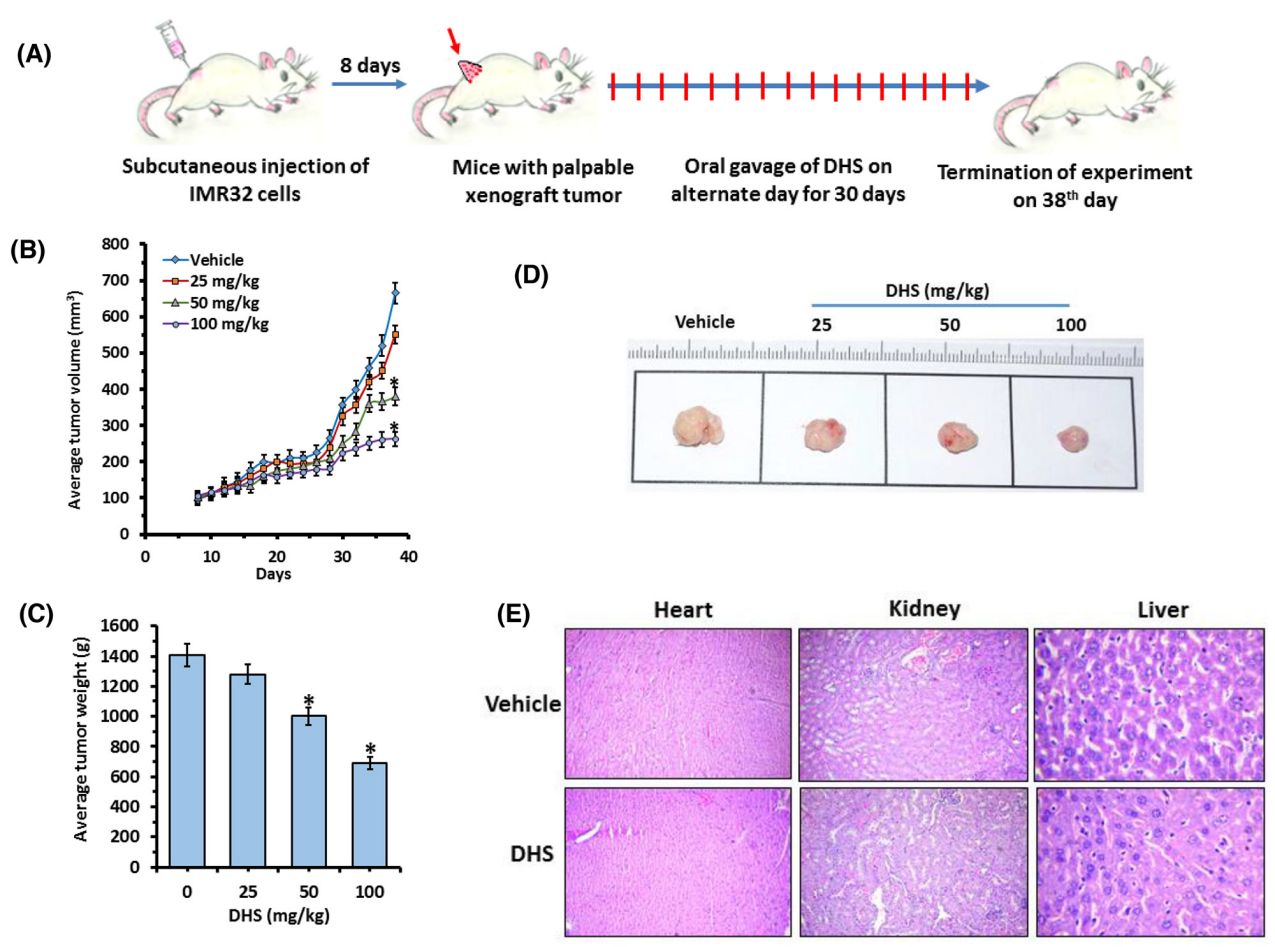
DHS inhibits neuroblastoma tumor xenograft growth in mice **(A)** Experimental protocol for tumor development and DHS treatment. **(B)** Average tumor volumes. **(C)** Tumor images of different treatment groups. **(D)** Average tumor weights. SCID mice bearing IMR32 neuroblastoma tumors were orally administered with vehicle alone or DHS (25, 50 and 100 mg/kg, single dose/day, alternate day during 8-38^th^ day of experiments). The tumor volumes were measured on every alternate day, while the tumor weights were measured on the 38^th^ day. The photographs of the tumors were captured with a digital camera on the 38^th^ day of the experiments, and representative photographs are shown. **(E)** Organ histology. Heart, kidney and livers were removed on the 38^th^ day of above experiments, the organ sections were stained with hematoxylin-eosin and analyzed with a bright-field microscope. The experiments were repeated three times with similar results, and the values are mean ± S. E. M. ^*^p<0.05 compared to vehicle-treated mice.

The IMR32 tumor volumes doubled in approximately 8 days and peaked up to ∼6 fold in the untreated control on the 30^th^ day from randomization (Figure 10A and 10B). Despite this, no tumor rupture or intraperitoneal bleeding was observed. The effect of DHS (50 and 100 mg/kg) was apparent on the 18^th^ day of treatment, when the tumor volumes started receding, compared to the control group (Figure 10B). At the end of the experiment (38^th^ day), DHS (50 and 100 mg/kg) reduced the mean tumor volumes by 30% and 51% respectively, compared to the control group (Figure 10B). DHS (25 mg/kg) was largely ineffective, reducing the mean tumor volume marginally (∼10%), compared to the control group. DHS also dose-dependently reduced the tumor weights, measured on the 38^th^ day (Figures 10C, 10D and Supplementary Figure 6A). In a separate experiment, we observed that DHS and Resv (100 mg/kg each) treatment reduced average tumor volumes by ∼51 and 29% respectively on the 38^th^ day of the experiment (Supplementary Figure 6B). No obvious toxicity, in terms of survival, activity and body weight was observed in the treatment groups, except for an initial slight (5%) weight loss following administration of DHS (100 mg/kg). No treatment-related diarrhoea or vomiting was observed, and the major organs did not show any abnormality on autopsy. The histological sections of liver, kidney and heart showed no gross changes in cellular architectures and morphology in DHS-treated *vis-à-vis* the vehicle- treated groups of mice (Figure 10E).

### DHS is non-toxic to rats

The chronic toxicity of DHS was evaluated from the behavioral changes, observed for 1 month as well as plasma biochemistry of the DHS (300 mg/kg)-fed rats. The animals had normal food and water intake as well as stool during the experimental period. The comparative plasma biochemistry profile (Table 2) of the normal rats and those treated with DHS revealed no hepatic and/or renal toxicity.

**Table 2:** Acute toxicity of DHS in rats^a^

parameter	normal rats	DHS-treated rats
Creatinine (mg/dl)	0.60 ± 0.08	0.53 ± 0.09
SGPT (U/l)	55.3 ± 5.5	59.7 ± 6.8
ALP (U/l)	104.2 ± 11.0	96.3 ± 8.1

^a^Each rat in the treatment group was given a single bolus dose of DHS (300 mg/kg) and the designated parameters in them as well as in the normal rats were estimated after a month. Each group contained 10 animals and the values are mean ± S. E. M.

## DISCUSSION

Although various hydroxystilbenes have shown encouraging anti-cancer property in pre-clinical/clinical studies, we disclose for the first time that amongst the tested hydroxystilbenes, DHS(compound **1**) was most effective, and showed better cytotoxic activity to both the neuroblastoma cell lines than Resv as reflected from the MTT and clonogenic assay results (Figures 1B–1D).

Apoptosis induction is an important cellular event, known to be mediated through (i) death receptors, *e. g*., Fas followed by activation of caspase-8, wherein mitochondria is involved only later; (ii) depolarization of MTP (*Ψ*_*m*_) leading to MMP mediated release of proteins such as cytochrome c, apoptosis inducing factor (AIF, a nuclease activator) and ApaF1 and subsequent activation of caspase-dependent or -independent pathways [8]. In the present study, the involvement of apoptosis (Figures 2A, 2C and 2D) and mediations of caspase initiator (caspase-9) and executor (caspase-3), but not of caspase-8 in the DHS-treated IMR32 cells (Figure 2E and 2F) was confirmed. Recently, several reports showed the mode of apoptosis induction by the stilbenes. Time dependent activation of caspases-8, -9 and -3 by Resv and pterostilbene was observed in many cancer cell lines [30, 31]. Compared to these results, the ability of DHS to induce caspase-8 independent apoptosis in the IMR32 cells may have clinical implications, given that loss of caspase-8 expression was reported in many patients, bearing malignant neuroblastomas [32,33]. Further, our results suggested that the intrinsic caspase-dependent mitochondrial apoptotic pathway may play a crucial role in the IMR32 cells in response to DHS (Figure 3A and 3B). Like the present results, earlier Resv induced mitochondria-mediated apoptosis in rat B103 neuroblastoma cells [34]. Nevertheless, mitochondria depletion could not completely abolish the DHS-induced apoptosis (Figure 3D), suggesting possible involvement of some non-mitochondrial pathway. Hence, we investigated the role of LMP as an alternative mechanism [22–24]. In addition, the possible inter-dependence of lysosomal destabilization and MMP was also investigated.

LMP mediated release of cathepsins can trigger the classical MMP-caspase pathway as well as MMP-dependent, but caspase-independent apoptosis [23]. Our data showed a DHS-mediated loss of lysosomal membrane integrity associated with cytosolic release of the active forms of CB, CD and CL (Figures 4A-4D), and inhibition of the cathepsins partially abrogated DHS-induced apoptosis and mitochondrial release of cyt. c in the IMR32 cells (Figure 4E and 9D). Previously, Resv induced death in cervical and colorectal cancer cells through lysosomal CD and CL respectively [35, 36]. However, in our experiments, inhibition of individual cathepsin types did not significantly alter the apoptosis (Supplementary Figure 4). All these data indicated that DHS-induced death might occur through LMP-mediated induction of MMP-caspase pathway or LMP-mediated caspase independent pathway.

Many BH3-only proteins from the BCL2/BAX family, when present locally in the mitochondrial membranes induce MMP. Upon apoptosis induction, cytosolic BAX moves into mitochondrial membranes and causes their permeabilization [8]. Likewise, cleavage of BID by the cathepsins [37] or caspase-3 [38] to the mitochondrially active, t-BID can induce the proapoptotic functionality of BAK and BAX, leading to cyt. c release. A previous report indicated that CB-dependent cleavage of the prominent MMP inducer protein, BID may emerge as a key connection between LMP and MMP [23]. In this context, DHS caused remarkable increase in BAX expression and BID cleavage, their translocation to mitochondria and lysosome along with decrease in the levels of BCL2 (Figure 5). These would increase the BAX/BCL2 ratio to favor apoptosis. Earlier, staurosporine treatment led to accumulation of BAX in human fibroblast lysosomes, leading to CD release into cytosol [39]. In a related study, t-BID was found to induce LMP in rat liver lysosomes and mouse embryonic fibroblasts without any involvement of BAX [40]. From these perspectives, the DHS-mediated lysosomal accumulation of both t-BID and BAX is an important finding of the present study, because the BAX-BID-silenced cells showed lesser LMP (Figure 9A and 9B) and reduced apoptosis (Figure 6B) *vis-à-vis* the SCR cells, on DHS treatment. The accumulated t-BID in the mitochondria and lysosome may assist oligomerization of BAX, sensitizing MMP in the DHS-treated cells. The significantly increased sensitivity of the BCL2-KD cells towards DHS and the reduced efficacy of DHS to the BAX-, BID- and BAX-BID-silenced cells confirmed the regulatory role of the BH3-only proteins in apoptosis (Figures 6A-6C).

Due to their higher metabolic rates, cancer cells have constitutively up-regulated ROS level that is targeted by many of the established chemotherapeutic drugs and the natural phenolics [41]. ROS can induce specific cellular responses through activation of the MAPKs signaling pathways [42]. Previous reports found that MAPKs are involved in the cytotoxicity of pterostilbene and Resv to tumor cells [31, 43], but the role of ROS and MAPKs in DHS-induced apoptosis of neuroblastoma cells has not been investigated so far. In this study, we observed generation of copious amounts of ROS by DHS, and pre-treatment of the cells with NAC abrogated the sensitivity of the cells to DHS treatment (Figures 7A-7C). Further, DHS treatment led to fast (within 4 h) activation of p38 and JNK MAPKs, but reduced the phospho-ERK 1/2 levels after an initial increase (Figure 8A). Also, the p38 and JNK inhibitors, but not the ERK 1/2 inhibitor reduced the sensitivity of the cells towards DHS treatment (Figures 8Bi-iii). Moreover, NAC pre-treatment prevented the DHS-induced p38 and JNK activation (Figure 8C), while cells, pre-treated with the p38 and JNK inhibitors did not show significant increase in BAX expression and BID cleavage (Figure 8D). Taken together, these results suggest that the DHS-induced cytotoxicity to the IMR32 cells is mediated through ROS-mediated activation of p38 and JNK, both of which were responsible for increased BAX expression and BID cleavage in the DHS-treated cells.

Presently, both LMP and MMP appear to be equally important in the cytotoxicity of DHS. Our JC-1 and LTR data showed occurrence of compromised mitochondrial and lysosomal membrane integrity at 3-4 h in the DHS-treated cells (Figure 3A and 4B), suggesting that DHS might have initiated LMP and MMP at the same time. Nevertheless, we hypothesized that LMP may be playing the central role on the following premises: (i) mitochondria depletion could not completely abolish the DHS-induced apoptosis; (ii) the observed protective effects of the caspase inhibitors may also be because of their ability block the lysosomal proteases at high concentrations [44]; and (iii) Stoka et al. reported that the leaked lysosomal proteases may cleave BID and induce MMP [38]. True to our expectations, compared to the SCR cells, DHS induced significantly less LMP in the BAX-, BID-, and BAX-BID-silenced cells (Figure 9A and 9B) that were also more resistant to DHS. Moreover, mitochondrial functional deficiency did not reduce the DHS-induced LMP in the cells at 16-24 h (Figure 9C). However, cells pre-treated with cathepsins inhibitors (an equimolar mixture of Pep A-Leu) showed reduced mitochondrial t-BID accumulation, cyt. c release and caspase-3 activation (Figure 9D).

**Figure 11:**
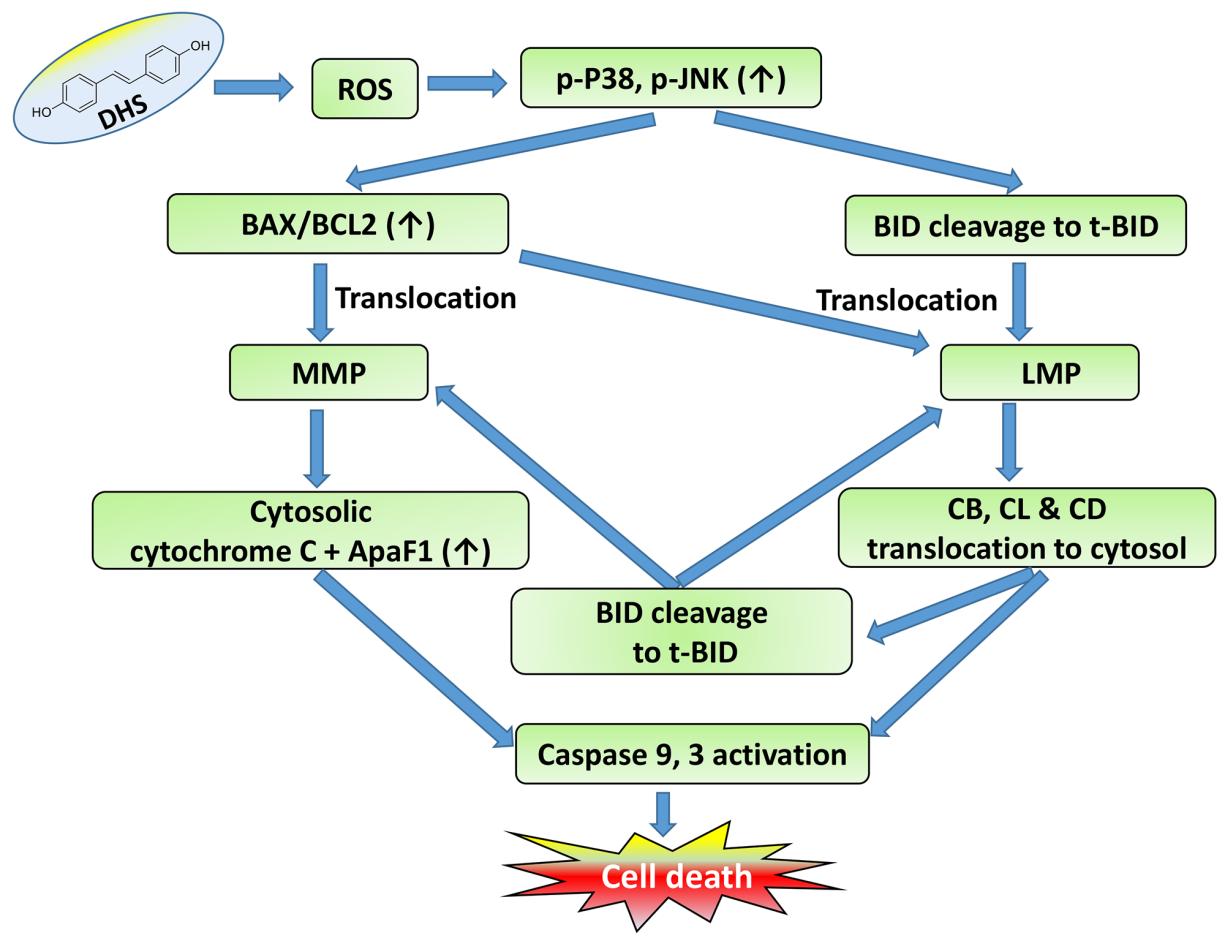
Schematic representation of the action of DHS

It is worth mentioning that the LMP and MMP initiation by DHS correlated reasonably well with BAX-translocation, t-BID accumulation and BCL2 down-regulation. DHS induced early translocations of BAX to the mitochondria that may account for the initial MMP in the cells. But, ROS generation, and BAX and t-BID translocation in the lysosome may be responsible for the observed LMP, caused by DHS. On the other hand, the delayed (24 h) translocation of t-BID in the mitochondria in response to DHS treatment (Figure 5B) may account for the LMP-induced MMP in the cells. Based on these results, it is tempting to propose that the lysosomal cathepsins-mediated alteration of BID cleavage (Figure 9D) sustains the mitochondrial pathway to apoptosis in the DHS-treated cells, at least partially. A schematic view of a proposed signaling network induced by DHS in the IMR32 cells to induce cell death is shown in Figure 11.

Finally we employed an ectopic implantation model in SCID mice to demonstrate the efficacy of DHS in reducing human neuroblastoma tumor burden. The growth kinetics data showed that almost with the onset of exponential tumor growth (28^th^ day of experiment), the inhibitory property of DHS (50 and 100 mg/kg) was evident and became very prominent with increasing treatment period (Figure 10B). Apparently the anti-tumor efficacy of DHS was slightly inferior to that reported with Resv using the NGP xenograft model [45]. However, this may be due to the use of a different neuroblastoma cell line, and also starting the drug treatment much earlier without allowing tumors to grow sufficiently in the previous study. This was also substantiated by the fact that DHS treatment (100 mg/kg) showed better results than Resv (100 mg/kg) in reducing IMR32 neuroblastoma in mice (Supplementary Figure 6B).

## CONCLUSIONS

In this work, we provided multiple evidences to show that DHS may be a potential, non-toxic chemotherapeutic option against neuroblastoma. The pharmacological action of DHS in the human neuroblastoma IMR32 cells was mediated through destabilization of mitochondrial and lysosomal membranes, associated with modulation of several related pro- and anti-apoptotic cascades of proteins. Several previous studies have shown that many of the anti-cancer compounds including Resv induce MMP and/or LMP sequentially to induce apoptosis in various cancer cell lines [36, 37, 45]. To the best of our knowledge, simultaneous MMP and LMP induction as well as LMP-mediated MMP induction by DHS in the IMR32 cells is absolutely novel. We believe that this is a key finding, as it provides options to tackle the problem of apoptosis-evasion, by targeting both mitochondria and lysosome. We have also shown that DHS is more effective than Resv against neuroblastoma cell lines. Our animal model study further implicated that oral administration of DHS for one month is well-tolerated, and has a greater therapeutic potential than Resv. Previously, DHS showed better pharmacokinetics than Resv in rats [46]. All these along with its non-toxicity in two different pre-clinical models (rat and mice) and ease of its synthesis in appreciable quantities, make DHS a new promising chemotherapeutic agent for human neuroblastoma.

## MATERIALS AND METHODS

### Chemicals and reagents

The hydroxystilbenes **1-5** as well as Resvwere synthesized and fully characterized as per the previous report [47]. Penicillin, streptomycin, phenylmethylsulfonyl fluoride (PMSF), aprotinin, sodium orthovanadate (NaVO_4_), cis-platin®, acridine orange (AO), Triton X-100, propidium iodide (PI), leupeptin (Leu) and pepstatin A (Pep A), RNAse A, 3-(4,5-dimethylthiazol-2-yl)-2,5-diphenyltetrazolium bromide (MTT), uridine, sodium pyruvate, N-acetylcysteine (NAC), ethidium bromide (EtBr), annexin-V kit, JC-1, 2’,7’-dichlorodihydrofluorescin diacetate (H2DCFDA), LYSISO1 kit, and antibodies for pro-caspases-3, -8, -9 were procured from Sigma chemicals (St. Luois, MO). Other chemicals used were: Dulbecco’s modified Eagle’s medium (DMEM), fetal bovine serum (FBS), Lysotracker Red (Life Technologies, Carlsbad, CA), Hoechst 33342 (Molecular Probes, Inc., Eugene, OR), Z-LEHD-FMK, Z-IETD-FMK, Z-DEVD-FMK, Z-VAD-FMK, U0126, SB203580 and SP600125 (Calbiochem, Gibbstown, NJ), antibodies for cytochrome c, cytochrome oxidase I (COX I), cytochrome oxidase IV (COX IV) and β-actin (Abcam Danvers, MA), for BAX, BCL-2, BID, t-BID, apoptosis-activating factor-1 (ApaF1), phosophorylated and non-phoshorylated -p38, -JNK, -ERK, cleaved caspases-3, -8, and -9 (Cell Signaling Technology Inc., Danvers, MA), for cathepsins-B, -L and -D (Novus Biologicals, Littleton, CO) and for LAMP1 (Santa Cruz Biotechnology Inc., Dallas, TX) and lipofectamine reagent (Invitrogen, Carlsbad, CA). Lumi-Light^PLUS^ western blotting kit and cell death detection^PLUS^ kit (Roche Applied Science, Baden-Wurttemberg, Mannheim) and nitrocellulose membrane (BioTrace® NT) from Pall Life Sciences (Easthills, NY) were also used. The antibodies used for cathepsin-B, -L and-D recognize the mature forms of the respective lysosomal enzymes.

### Cell culture

The IMR32 (human CNS-derived neuroblastoma), SHSY-5Y (human PNS-derived neuroblastoma), INT407 (human normal intestinal), and HEK293 (human normal kidney) cell lines were procured from National Centre for Cell Sciences, Pune, India. The cells were routinely seeded at a density of 0.1-5 × 10^6^ cells/ml and grown in DMEM medium supplemented with 10% heat-inactivated FBS, 2 mM glutamine, 100 U/ml penicillin, and 100 mg/ml streptomycin in a humidified 5% CO_2_ atmosphere at 37 °C. Cells were passaged every 2-4 days to maintain 80-90% confluency. The cell viability was determined by the trypan blue dye exclusion assay. Subcultures were obtained by trypsinization with 0.25% trypsin in phosphate buffered saline (PBS).

### Animals

Male Wistar rats (6-10 weeks,300-330 g), bred at the BARC laboratory animal house facility, Mumbai, India, were procured after obtaining clearance from the BARC Animal Ethics Committee (BAEC/14/08 dt. 05/09/2009). All animals were individually housed in polycarbonate cages in an air-conditioned room (temperature: 23 ± 1°C, relative humidity: 55 ± 5%, 12-h automatic lighting: 07:00-19:00 hrs) and given food pellets as per National Institute of Nutrition (NIN), Hyderabad, India and tap water ad libitum.

Severe combined immune-deficient (SCID) mice were procured from VivoBioTech, Hyderabad, India after obtaining clearance from the BARC Animal Ethics Committee (BAEC) (Approval No. BAEC/20/15 dt. 14/09/2015). The mice (4-5 weeks old, 20–25 g) were reared on a balanced laboratory diet as per National Institute of Nutrition (Hyderabad, India), and given tap water ad libitum. They were kept in well-ventilated cages at 20 ± 2 °C, 65–70% humidity, and day/night cycle (12 h/12 h). All animals were handled following International Animal Ethics Committee Guidelines, and the experiments were permitted by BAEC. All the experiments were conducted with strict adherence to the ethical guidelines laid down by the European Convention for the Protection of Vertebrate Animals used for Experimental and Other Scientific Purposes. In addition, the ethical guidelines laid down by the Committee for the Purpose of Control and Supervision of Experiments on Animals, constituted by the Animal Welfare Division, Government of India, on the use of animals in scientific research were followed. The body weights, and food and water intakes of the rats were measured daily at 9.00 AM during the entire experimental period.

### Sample preparation

Freshly prepared stock solutions of compounds **1-5** and Resv (20 mM) in DMSO were diluted with DMEM to attain the required concentrations, and used for the cell line studies. For the mice experiments, DHS solutions were prepared in Neobee M5 oil (Sigma) to attain the required concentrations.

### MTT assay

The MTT reduction assay was carried out using a reported protocol, with minor modifications [48]. Briefly, the IMR32 and SHSY-5Y cells (5 × 10^3^/well) grown in 96-well plates and treated with different concentrations of the compounds **1-5** and Resv for 48 h were washed once with PBS, MTT solution (0.5 mg/ml, 100 μl) added to each well and kept at 37 °C for 6 h. The formazan crystals in the viable cells were solubilized with 0.01 N HCl (100 μl) containing 10% SDS and the absorbance at 550 nm read using a spectrophotometric plate reader (Bio-Tek, MQX 200, VT, USA). Additional MTT assays were carried out after incubating the IMR32 and SHSY-5Y cells with a wider range of concentrations of DHS and Resv for 24 and 48 h to obtain their respective IC_50_ values. Similar experiments were also carried out using IMR32 cells, pretreated with NAC (5 mM) for 1 h, followed by incubation with DHS (0-50 μM) for 24 and 48 h. NAC was present throughout the experiments.

### Clonogenic survival assay

The cells (1 × 10^3^/well) were seeded in 6-well plates and incubated with vehicle (0.1% DMSO) or different concentrations of DHS (0-5 μM) or Resv (0-15 μM) at 37 °C for 24 h. The medium was removed, the untreated and treated cells were washed with PBS, and incubated at 37 °C for 12 d in the growth media, replacing the media every 3 day. The colonies were fixed with methanol and stained with 0.5% crystal violet in 1:1 methanol-water. Colonies were counted, and images of colonies were scanned. The surviving fractions were determined from the colony counts and corrected for the plating efficiency (78.3%) of the non-treated controls.

### Fluorescence microscopy

The IMR32 cells (5 × 10^5^ cells/well), cultured on cover slips in 6-well plates were incubated with DHS (0, 20 and 40 μM) for 48 h. The cells were stained with Hoechst 33342 (10 μM) for 15 min, washed once with PBS, mounted with 70% glycerol, and analyzed under an Axioskop II Mot plus (Carl Zeiss, Germany) microscope (40 × optics). For each treatment, minimum 100 cells were manually observed and the number of cells with fragmented and condensed nuclei counted.

### Flow cytometry

All the flow cytometry analyses were carried out with a Pertec CyFlow® Space flow cytometer using the FlowJo software. Cellular debris was excluded from the analyses by raising the forward scatter threshold. At least 2 × 10^4^ cells of each sample were analyzed, and the data were registered on a logarithmic scale.

### Apoptosis analyses

The hypodiploid DNA content (sub-G1) were analyzed as a marker for apoptosis by flow cytometry. Briefly, the IMR32 cells (5 × 10^5^ cells/well), treated with different concentrations of DHS or Resv for 24 and 48 h were collected, washed with cold PBS and incubated in PBS containing Triton X-100 (0.1%), PI (50 μg/ml) and RNAse A (100 μg/ml) for 30 min at 37 °C, and analyzed by flow cytometry. The apoptotic nuclei appeared as broad hypodiploid DNA peaks. Similar experiments were also carried out using cells, pre-treated with inhibitors of caspase-9, caspase-8, caspase-3, pan-caspase, p38, ERK, JNK, cathepsins (Leu, Pep A, individually or in combination) respectively for 1 h, followed by incubation with DHS (20 μM). Additional sub-G1 assays were carried out with BAX-KD, BCL2-KD, BID-KD, BAX- BID-DKD and *ρ*^o^ IMR32 cells. The flipping of PS was assessed by using the annexin V/PI apoptosis detection kit as per the manufacturer’s instructions. Briefly, the untreated and DHS (20 μM)-treated IMR32 cells (5 × 10^5^/well) were incubated for different periods, the cells were washed twice with cold PBS, suspended in a binding buffer (100 μl), stained with annexin V (1 μl) and PI (5 μl). The percentage of annexin V positive and PI negative cells in the total cell population was analyzed.

### Analysis of mitochondrial and lysosomal membrane integrity

The loss of *ΔΨ*_*m*_ [49], lysosomal membrane integrity and LMP [50] were assayed using well established reported protocols, with minor modifications. Briefly, the IMR32 cells (5 × 10^5^ cells/well), cultured in 6-well plates were incubated with DHS (0 and 20 μM) for 0-24 h, washed with PBS, and incubated with JC-1 (20 μM), AO (10 μM) or LTR (100 nM) for 15 min at 37 °C. The cells were collected, washed two times with cold PBS and analyzed by flow cytometry. The *ΔΨ*_*m*_ loss was quantified from the shift of JC-1 emission from red (∼590 nm, channel Fl3) to green (∼525 nm, channel Fl1), while the lysosomal membrane integrity was quantified from the percentage of cells (Fl3 subset) showing reduced red florescence of AO or LTR.

### Intracellular ROS level assay

The IMR32 cells were incubated with DHS (0 and 20 μM) for different time periods. After addition of H2DCFDA (10 μM), the cells were incubated for 30 min at 37 °C, collected by trypsinization, washed two times with PBS, and resuspended in PBS. The ROS levels, expressed in arbitrary units were analyzed from the increased green fluorescence (excitation at 480 nm and emission at 530 nm) of oxidized DCFDA by flow cytometry [51]. H_2_O_2_ (200 μM) was used as the positive control and incubation was carried out for 2 h.

### Generation of mitochondria-deficient IMR32-*ρ*^*o*^ cells

The mitochondria-deficient IMR32-*ρ*^*o*^ cells were generated and maintained as described previously [52] with minor modifications. Briefly, the IMR32 cells were maintained in a complete medium supplemented with 1 mM sodium pyruvate, 1 mM uridine and 50 ng/ml EtBr (24 passages for 8 weeks). The cells, cultured in medium without EtBr served as the control (wild-type *ρ*^***+***^-IMR32). Mitochondria depletion in the *ρ*^*o*^-IMR32 cells was confirmed by analyzing the loss of mitochondria specific protein, COX I and mitochondrial DNA as per the reported protocol [53].

### Western blotting

The IMR32 cells were incubated with DHS (0 and 20 μM) for different time periods and lysed in a lysis buffer (20 mM pH 7.4 Tris, 250 mM NaCl, 2 mM pH 8.0 EDTA, 0.1% Triton X-100, 0.01 μg/ml aprotinin, 0.01 μg/ml Leu, 0.4 mmol/l PMSF, and 4 mmol/l NaVO_4_) to obtain whole cell extracts. The lysates were spun at 16500 × g for 10 min, the supernatants collected and kept at -70 °C. In separate experiments, the mitochondrial and cytosolic extracts were prepared as described [54]. The lysosomal extract was prepared using the LYSISO1 kit, following manufacturer’s protocol. The whole cell extracts of the cells pre-treated with NAC (5 mM) or different inhibitors for 1 h, followed by incubation with DHS (0 and 20 μM) for different times were also prepared.

The cellular and sub-cellular fractions were individually separated by 10-15% SDS-polyacrylamide gel electrophoresis, and electrotransferred to nitrocellulose membrane. The membranes were blocked for 1 h at room temperature in TBST buffer (20 mM Tris–HCl, pH 7.6, 137 mM NaCl, and 0.1% Tween-20) containing 5% (w/v) nonfat milk, and then incubated overnight at 4 °C with the required specific primary antibodies. After several washes, suitable HRP-conjugated secondary antibodies were added, the membranes incubated further for 2 h, and the blots of various proteins were developed using a Lumi-Light^PLUS^ western blotting kit. In order to probe phosphorylated and non-phosphorylated forms of the MAPKs on the same blot membrane, standard stripping protocol was used. The bands were detected using a Kodak Gel-doc software and the intensity ratios of the individual bands to that of normal control, taken as 1 (arbitrary unit) were quantified after normalizing with respect to the loading controls.

### Transient and stable RNA interference in IMR32 cells

Following manufacturer’s protocol, the cells were transfected with short interfering RNA (siRNA) purchased from Santa Cruz Biotechnology, Inc. (CA, USA) encoding siRNA specific for BAX, BID or the nonspecific control by using lipofectamin RNAI MAX transfection reagent to generate BAX-KD, BID-KD and BAX-BID-DKD or respective control SCR cells. The nonspecific control siRNA contained a scrambled sequence with no significant homology to rat, mouse, and human gene sequences and are designated as SCR cells. The transfected cells were grown for 48 h in a transfection medium and the expressions of BAX and BID were assessed by immunoblots.

In another set of experiments, the cells were transfected with plasmids (Imgenex, Sandiego, CA) encoding scrambled short-hairpin RNA (shRNA) to generate control SCR cells or shRNA against BCL2 to generate BCL2-KD cells, using lipofectamine 2000. The BCL2-KD, and the respective SCR cells were grown for two weeks in a medium containing G418 (800 μg/ml). Several antibiotic-resistant clones were expanded and screened for the BCL2 protein. The clones with the lowest expression were selected for the studies and maintained further in the presence of G418 (800 μg/ml).

### Ectopic tumor xenograft and toxicity studies in a preclinical mice model

Male SCID mice were xenografted at the age of 6 weeks (mean body weight, 22.4 g). The IMR32 human neuroblastoma cells were grown up to 70% confluency, harvested by trypsinization, washed twice with ice-cold DMEM. The cells were re-suspended in ice-cold DMEM and kept on ice until xenotransplantation. Right flank region of the male recipient SCID mice were cleansed with 70% ethanol and subcutaneously injected with IMR32 cells (1 × 10^7^ cells/0.2 ml/mouse). After eight days, mice with palpable tumors were randomized and divided into four groups (8 mice/group). The treatment groups received DHS (25, 50, 100 mg/kg, single dose/day, alternate day during 8-38^th^ day of experiments) by oral gavage. The control group received vehicle (Neobee M5 oil, 0.2 ml) only throughout the experimental period (8-38^th^ day of experiments). Additional experiments were also carried out with Resv or DHS (each 100 mg/kg), under the above experimental conditions. The tumor growth was monitored three times a week by calipers and the tumor volumes calculated according to the formula 0.5 × length × width2. Four h after the last dose of the treatments on the 38^th^ day of the experiments, the mice were sacrificed after an overdose of thiopental, the tumor xenografts were removed and their volumes measured. The animals were dissected and macroscopic analysis was also carried out to visualize major morphological changes in the organs. The photographs tumor bearing mice and excised tumors were captured with a digital camera (Nikon D5500). These experiments were repeated three times. For the histopathological study, liver, kidney and heart samples were fixed in Bouin’s fixative, 5 μm thick paraffin sections were prepared and stained with hematoxylin–eosin (H–E). The stained slides were analysed under a bright-field light microscope (Axioskop II Mot plus, Carl Zeiss, Germany).

### Toxicity studies in rat model

A stock solution of DHS (100 mg/ml) in DMSO was diluted with edible oil and an appropriate volume, amounting to 300 mg/kg of DHS was given to the rats by oral gavage (a single bolus dose). The serum samples were collected after a month and the liver and kidney function parameters analyzed with an auto analyzer (Rx Daytona, Randox, crumlin county, Antrim, UK). The normal untreated rats served as the control. Each group contained 10 animals.

### Statistical analysis

All determinations were made in five replicates in 3-4 different experiments and the values are mean ± S.E.M. The data were analyzed by paired t-test and one-way analysis of variance (ANOVA) followed by a Dunnett multiple comparisons post-test. A probability value of *p*<0.05 was considered significant. The vehicle-treated cells were considered as the untreated control.

## SUPPLEMENTARY MATERIALS FIGURES


