# Supplementary material for: trans-4,4’-Dihydroxystilbene (DHS) inhibits human neuroblastoma tumor growth and induces mitochondrial and lysosomal damages in neuroblastoma cell lines
Source: Oncotarget. 2017 May 16;8(43):73905–24. doi: 10.18632/oncotarget.17879 (PMC5650311; doi:10.18632/oncotarget.17879)
Supplement: Supplementary file 1 [file oncotarget-08-73905-s001.pdf]

trans-4,4′-Dihydroxystilbene (DHS) inhibits human neuroblastoma tumor growth and induces mitochondrial and lysosomal damages in neuroblastoma cell lines

SUPPLEMENTARY MATERIALS

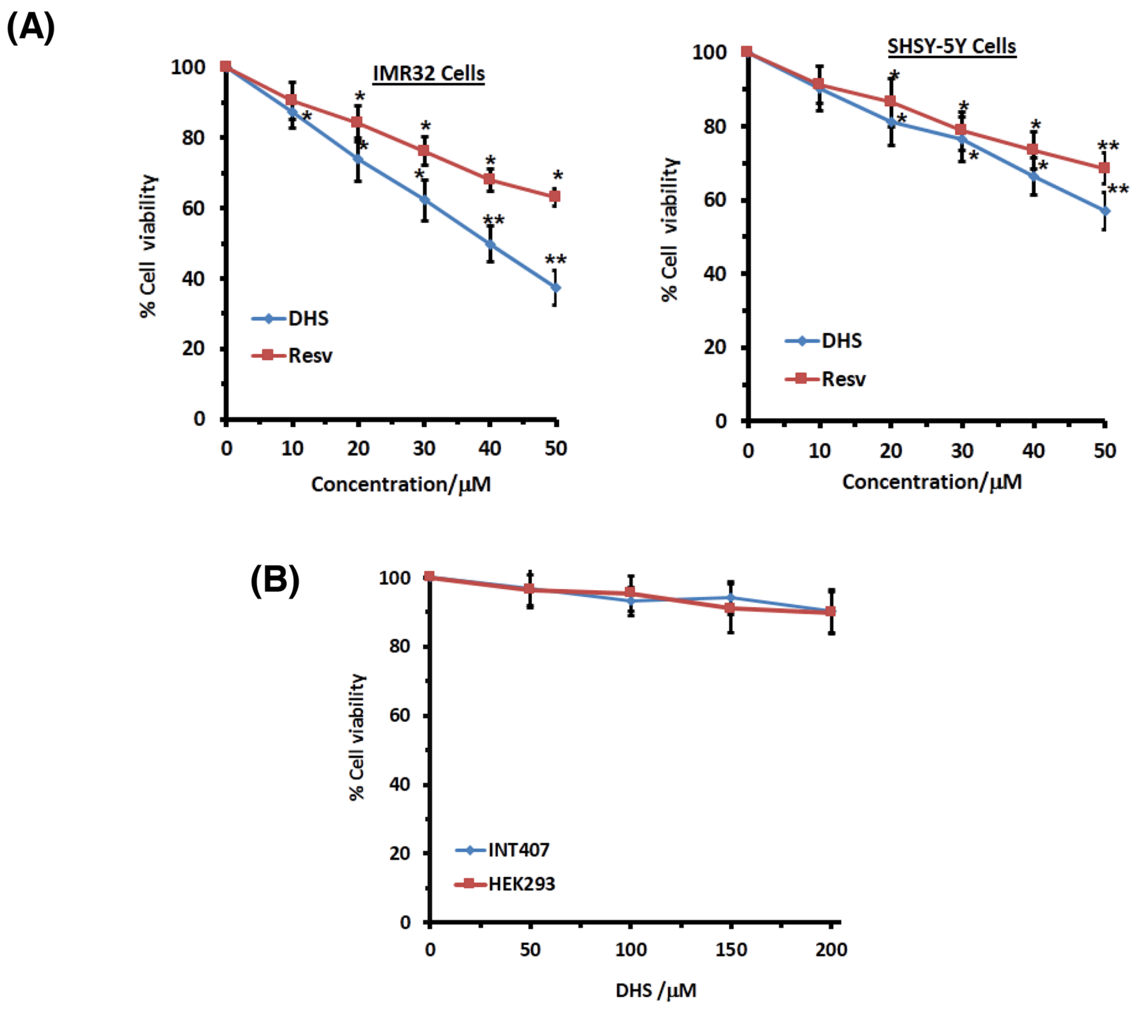

Supplementary Figure 1: Dose-dependent cytotoxicity of DHS and Resv against IMR32, SHSY-5Y cells, and effect of DHS on INT407 and HEK293 cells. **(A)** Cytotoxicity to neuroblastoma cell lines. The cells were incubated with vehicle (0.1% DMSO) or increasing concentrations of DHS or Resv. Cell viability at 24 h was assessed by the MTT assay. **(B)** Non-toxicity of DHS to INT-407 and HEK-293 cell lines. The cells were incubated with vehicle (0.1% DMSO) or DHS (50-200 µM) and the cell viability at 48 h was assessed by the MTT assay. All determinations were made in five replicates in 3-4 different experiments and the values are mean ± S.E.M. $^{*}p$<0.05, $^{**}p$<0.01 compared to vehicle control.

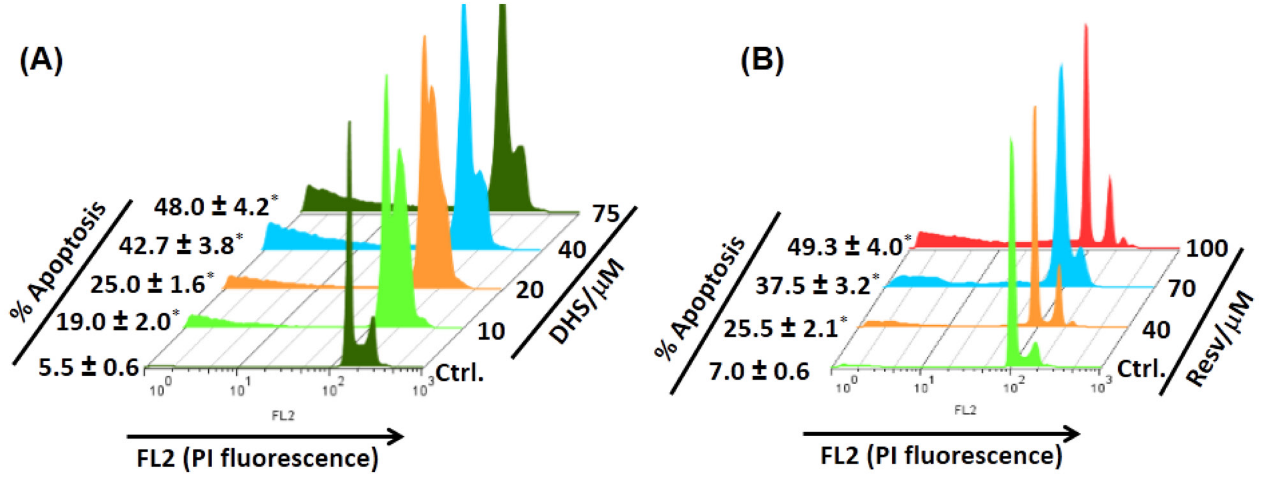

Supplementary Figure 2: Dose-dependent apoptosis induction by DHS and Resv in IMR32 cells. The cells were incubated with different concentrations of DHS **(A)** or Resv **(B)** for 24 h, and the sub-G1 cell population analyzed by flow cytometry. All determinations were made in five replicates in 3-4 different experiments and the values are mean ± S.E.M. $^{*}p<0.05$ compared to vehicle control.

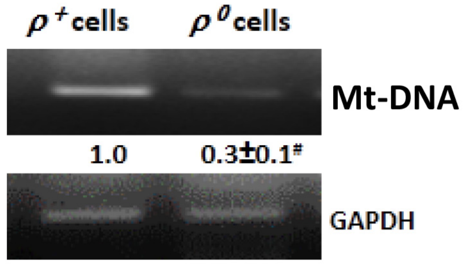

Supplementary Figure 3: MtDNA assessment in IMR32-ρ^{+} and ρ^{o} cells. The mitochondrial DNA levels in the IMR32-ρ^{+} and ρ^{o} cells were assessed by PCR. The EtBr stained DNA bands in the agarose gel were detected using a Kodak Gel-doc software and the intensity ratios of the individual bands to that of IMR32-ρ^{o} control, taken as 1 (arbitrary unit) were quantified after normalizing with respective loading controls. The determinations were made in duplicates in 3 different experiments. Representative images are shown.

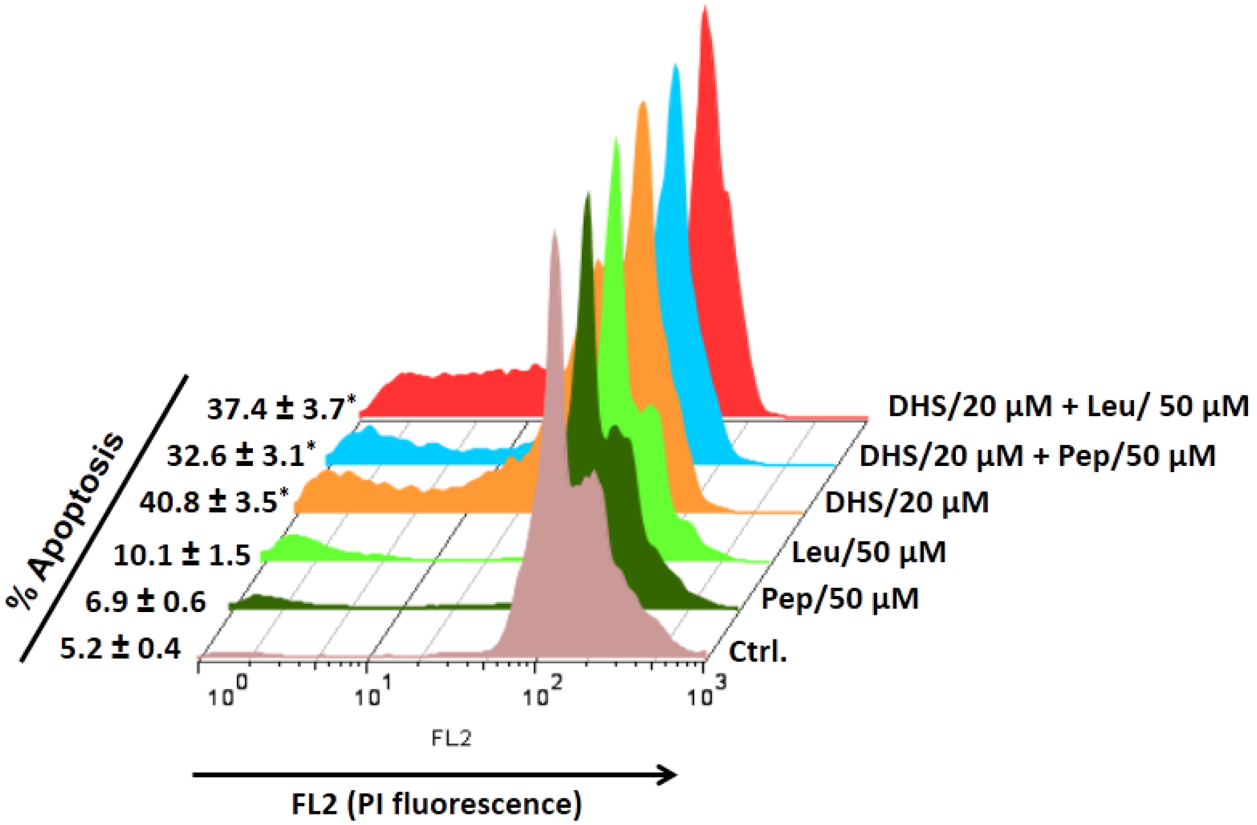

Supplementary Figure 4: Effects of individual cathepsins inhibitors on the DHS-induced apoptosis in IMR32 cells. The cells were treated with vehicle (0.1% DMSO), Pep A or Leu for 1 h followed by incubation with DHS (0 and 20 µM) for 48 h, and the sub-G1 cell populations analyzed. All determinations were made in five replicates in 3-4 different experiments and the values are mean ± S.E.M. $^{*}p<0.05$ compared to vehicle control.

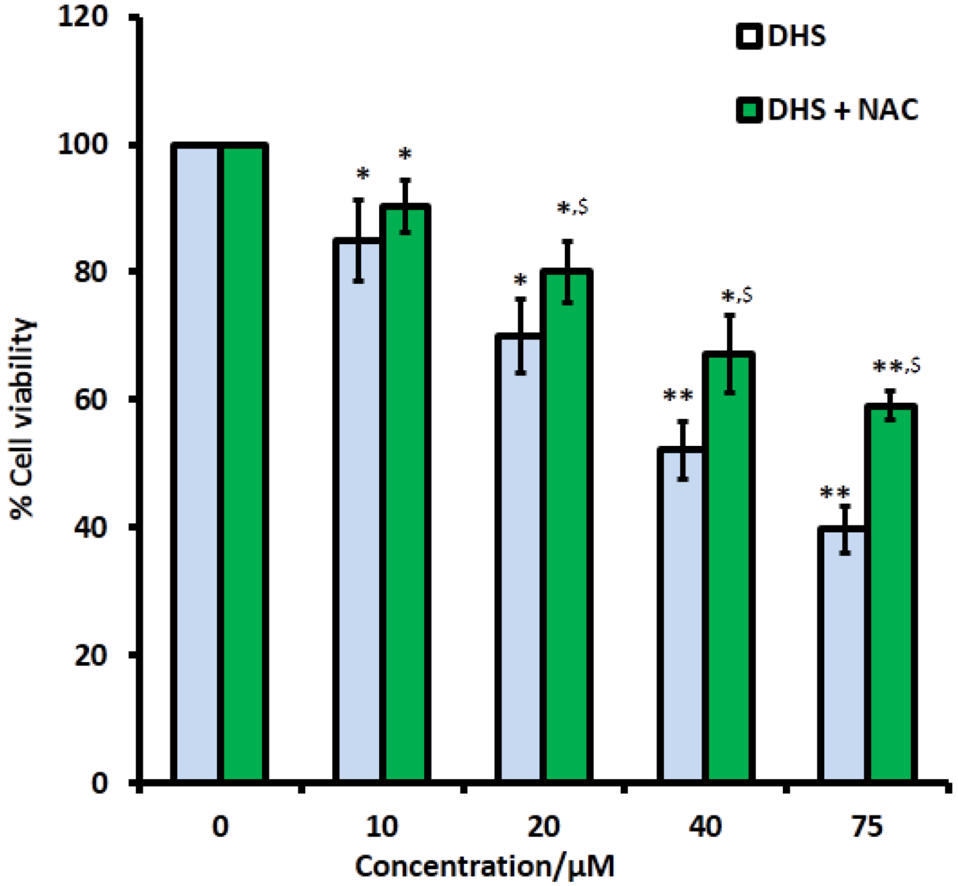

Supplementary Figure 5: Effect of NAC on survival of IMR32 cells after DHS treatment. The cells were pretreated with NAC (5 mM) for 1 h, further incubated for 24 h with DHS and the cell viability was assayed by MTT reduction method. All determinations were made in five replicates in 3-4 different experiments and the values are mean ± S.E.M. $^{*}p<0.05$ $^{**}p<0.01$ compared to vehicle control; $^{\$}p<0.05$ compared to only DHS treatment.

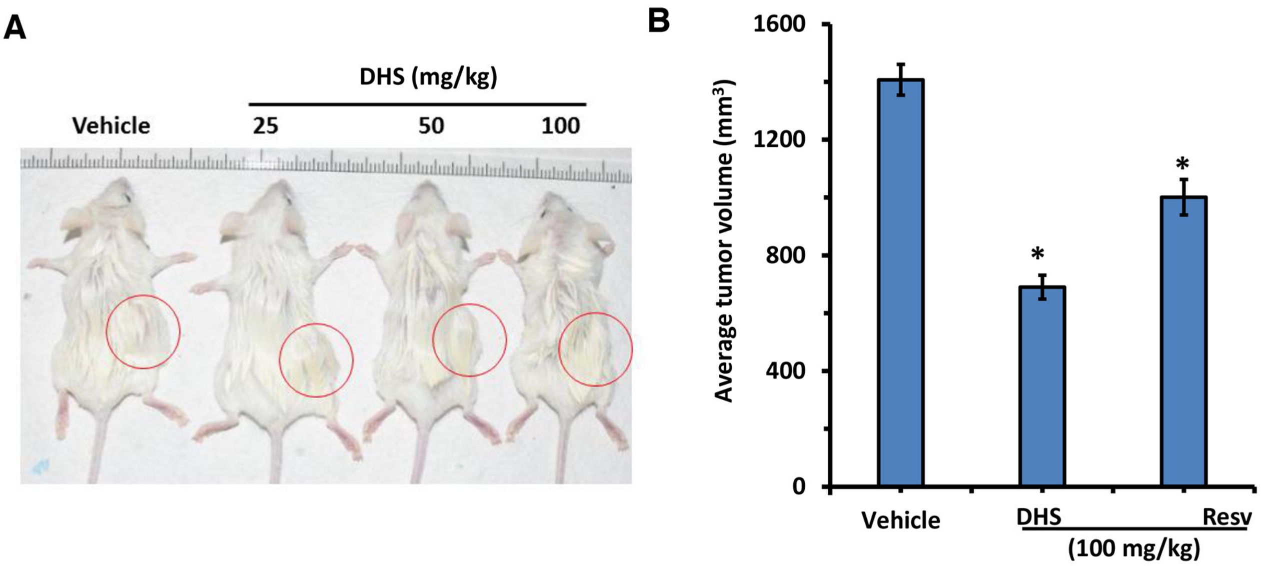

Supplementary Figure 6: DHS inhibits neuroblastoma tumor xenograft growth in mice. (A) Photographs of neuroblastoma tumor-bearing untreated and DHS-treated mice. SCID mice bearing IMR32 neuroblastoma tumors were orally administered with vehicle alone or DHS (25, 50 and 100 mg/kg, single dose/day, alternate day during 8-38th day of experiments). The photographs of all the mice were captured with a digital camera on the 38th day of the experiments, and representative photographs are shown. **(B)** Relative efficacy of DHS and Resv in reducing tumor volumes of neuroblastoma tumors in mice. SCID mice bearing IMR32 neuroblastoma tumors were orally administered with vehicle alone, DHS or Resv (100 mg/kg each) as above. The tumor volumes were measured on the 38th day. The experiments were repeated three times with similar results, and the values are mean ± S. E. M. $^{*}p<0.05$ compared to vehicle-treated mice.
